# The OJIP Kinetics Analysis Reveals Differential Thermal Tolerance Responses in Photosystem II of *Coffea canephora* Clones After Two Recurrent Cycles of Water Deficit

**DOI:** 10.3390/plants15050740

**Published:** 2026-02-28

**Authors:** Guilherme Augusto Rodrigues de Souza, Danilo Força Baroni, Diesily Andrade Neves, Anne Reis Santos, Laísa Zanelato Correia, Larissa Crisostomo de Souza Barcellos, Ellen Moura Vale, Wallace de Paula Bernado, Weverton Pereira Rodrigues, Antelmo Ralph Falqueto, Miroslava Rakocevic, Eliemar Campostrini

**Affiliations:** 1 Plant Physiology Laboratory (LMGV), State University of North Fluminense Darcy Ribeiro (UENF), 2000 Alberto Lamego Ave., Parque Califórnia, Campos dos Goytacazes 28013-602, RJ, Brazil; baronidf@gmail.com (D.F.B.); diesilyandrade@gmail.com (D.A.N.); annersantos@outlook.com (A.R.S.); laisazanelatocorreia@gmail.com (L.Z.C.); lbarcellos.uenf@gmail.com (L.C.d.S.B.); ellenmoura27@gmail.com (E.M.V.); campostenator@gmail.com (E.C.); 2 Instituto de Biodiversidade e Sustentabilidade, Universidade Federal do Rio de Janeiro (UFRJ), 764 Amaro Reinaldo dos Santos Silva Ave., São José Barreto, Macaé 27965-045, RJ, Brazil; 3 Sylvio Moreira Citrus Research Center-Agronomic Institute (IAC), Rod. Anhangüera, km 158, Cascalho, Cordeirópolis 13490-000, SP, Brazil; wallace-bernardo@hotmail.com; 4 Centro de Ciências Agrárias, Universidade Estadual da Região Tocantina do Maranhão (UEMASUL), 100 Agrária Ave., Res. Colina Park, Imperatriz 65900-001, MA, Brazil; wevertonuenf@hotmail.com; 5 Departamento de Ciências Agrárias e Biológicas, Universidade Federal do Espírito Santo (UFES), BR 101 Norte, km 60, Bairro Litorâneo, CEP, São Mateus 29932-540, ES, Brazil; antelmofalqueto@gmail.com; 6 Laboratory of Crop Physiology, Department of Plant Biology, Institute of Biology, State University of Campinas (UNICAMP), Campinas 13083-862, SP, Brazil

**Keywords:** Conilon coffee, heat stress, photochemical damage, priming, stress memory

## Abstract

*Coffea canephora* cultivation areas in Brazil are frequently exposed to successive cycles of water deficit, triggering plant stress responses. In addition to water deficit, increased air temperature can act as a second stress factor. The recurrence of these stress factors may induce plant tolerance mechanisms, potentially mitigating future stress responses even of a different stress nature. We hypothesized that repeated cycles of water deficit can trigger tolerance mechanisms that make *C. canephora* leaves more resilient to supra-optimal temperatures. To test this hypothesis, young *C. canephora* plants were grown under non-limited water conditions for seven months (Ψ_mSoil_ > −20 kPa), after which they were subjected to two consecutive cycles of water deficit (Ψ_mSoil_ < −300 kPa), followed by rehydration. Two clones were used, ‘A1’ and ‘3V’, previously classified as drought sensitive and tolerant, respectively, considering the dynamics of physiological and architectural responses. After the second cycle, leaf discs were collected from completely expanded leaves formed during the two stress cycles and exposed to heat treatments (35 °C, 40 °C, 45 °C, 50 °C, and 55 °C) for 15 min in a water bath. Chlorophyll *a* fluorescence emission was then monitored, and the results were analyzed using OJIP transient kinetics and the JIP_Test_. High temperatures induced negative changes in both OJIP kinetics and JIP_Test_-derived parameters. A significant increase in *F*_0_ and a reduction in *F*_M_ were observed mainly at 50 °C and 55 °C, due to changes in the stages of the OJIP curve. These changes impacted the “energy connectivity” and consequently the electron transport along the electron transfer chain (ETC), increasing energy dissipation, as confirmed by the JIP_Test_ variables. Despite the high temperature impacts, previous water deficit induced heat tolerance in clone ‘A1’, while it increased sensitivity in clone ‘3V’. This study suggests that selecting drought-resistant varieties should consider their subsequent response to short high-temperature stress to avoid cross-sensitivity caused by selecting for a single environmental factor.

## 1. Introduction

*Coffea canephora* Pierre ex A. Froehner (robusta coffee) is the second most cultivated and consumed coffee species worldwide. Brazil ranks second in the production and export of grains of robusta coffee [1]. The primary contributor to Brazilian *C. canephora* production is the Espírito Santo state, which accounts for approximately 70% of the national output [2]. Productivity in this region has faced ongoing challenges due to water scarcity and rising average air temperatures, particularly in non-irrigated crops [3]. Numerous studies have highlighted the negative impacts of drought and supra-optimal temperatures on the cultivation of *Coffea* spp., mainly due to stomatal and non-stomatal photosynthetic limitations [4,5,6,7].

Supra-optimal temperature stress directly damages the photosynthetic apparatus due to the sensitivity of photosystem II (PSII) reaction centers to heat [8]. High temperatures reduce the stability of the Mg cluster in the oxygen-evolving complex (OEC) [9], thereby limiting the electron supply to the electron-transport chain (ETC) [10]. Additionally, increased membrane fluidity results in a decoupling of electron acceptors, leading to a reduction in electron flow towards photosystem I (PSI), which restricts the synthesis of ATP and NADPH [11]. In *Coffee* spp., heat stress decreases photochemical efficiency [12], reduces the electron transport rate and the activity of photosynthetic and respiratory enzymes [5,13], and limits/inhibits the photosynthetic assimilation rate [5,14]. The extent of damage resulting from heat stress and the ability of plants to recover after stress relief are strongly impacted by the genotype and the leaf age [15,16] and by the combination of stressors, including drought [5,17].

The simultaneous occurrence of multiple stress factors, such as water scarcity combined with supra-optimal temperatures, can severely damage the photosynthetic apparatus and reduce crop productivity [18]. The recurrence of stressful conditions can trigger stress responses that activate certain tolerance mechanisms in plants [19,20,21]. These acclimatization mechanisms are associated with plant “stress memory” and are being studied for the development of cultivation techniques, such as priming, which enhances plants’ ability to cope with environmental stress [22]. Repeated drought cycles in *Coffea* spp. can trigger physiological adjustments that confer increased tolerance to future water deficits [6,21,23], and the occurrence of a previous water deficit can induce tolerance to heat stress [24]. The regulation of antioxidant protection, metabolic reprogramming—involving hormone metabolism, osmoregulators, protective metabolites, lipid and fatty acid metabolism—as well as molecular responses and epigenetic changes, may all contribute to the acquisition of heat tolerance [24]. In crop species, such as common bean [25], wheat [26], or maize [27], as well as in species from natural environments, such as tropical forest trees [28] or alpine grasses [29], plants frequently exposed to drought may develop enhanced thermotolerance induced by drought events, characterized by “stress memory” responses. Such phenomena occur because the signaling pathways underlying drought and heat stress responses are shared, as these conditions commonly occur simultaneously [30]. It was shown that PSII stability increased after previous exposure to environmental pressures [31,32]. However, studies demonstrating the acquisition of drought-induced thermotolerance remain scarce for most agricultural crops.

In recent decades, the occurrence and severity of various types of environmental stresses on plants have been monitored in a practical, fast, and non-invasive way using chlorophyll (Chl) *a* fluorescence (ChlF) emission [33,34,35]. The use of the direct fluorescence technique allows measuring the “fast kinetics” of Chl *a* fluorescence between the O (initial fluorescence) and the P (maximum fluorescence) steps of the induction curve [36]. This is the fluorescence transient (induction), occurring due to changes in the various photosynthetic reactions and their regulation. When a ChlF induction curve is plotted on a logarithmic scale between the O and the P steps (between 0 and 1000 ms), two inflection points become evident, known as J (2–3 ms) and I (20–30 ms) steps, which is why it is commonly called the “OJIP curve” [36]. By analyzing the kinetics of the OJIP transient and its changes, it is possible to evaluate particular PSII-related biophysical events that describe the energy flow from light energy absorption and electron transport in the PSII reaction centers (RCs) to the reduction of the final PSI electron acceptors. The peak that appears around 300 µs between the O and K steps (L-band) indicates decreased energy transfer between PSII antenna complexes, suggesting that the system is vulnerable to damage, serving as an indicator of short-term stress [37,38,39]. The rise in the ChlF induction curve between the O and J steps is mainly related to the primary photochemical stage, meaning the occurrence of a single turnover of the Q_A_ reduction event [37,38,39]. In it, the primary quinone electron acceptor Q_A_ is reduced only once. The fluorescence rise between the J and I steps reflects the capacity of the system to reduce electron acceptors between the PSII and PSI, such as Q_B_, plastoquinone (PQ), cytochrome b6/f (cyt), and plastocyanin (PC) [37,38,39,40]. The J–I phase is strongly related to the partial reduction of PQ [40]. The fluorescence rise from J to P steps is related to the multiple turnovers of PSII reduction until achieving complete closure of the RCs, where biochemical responses predominate [37,38]. Finally, the ChlF rise between I and P is mainly related to the reduction of final PSI acceptors, especially ferredoxin (FD) and NADP^+^ [37,38,39]. In some cases, the occurrence of drought or thermal stress can trigger the appearance of another step, the K-band between O and J steps, which is directly related to OEC damage [33,41] or to changes in the functional size of the PSII antenna complex [42]. In addition to OJIP kinetics analysis, numerous other quantitative parameters can be derived through the JIP_Test_ analysis, based on the theory of energy flow in biomembranes [43].

We hypothesized that *C. canephora*, commonly cultivated in hot regions and frequently exposed to drought events [3], may exhibit “stress memory” responses associated with the acquisition of thermotolerance. Furthermore, we expected that this acquired tolerance could be interpreted through the analysis of OJIP kinetics and JIP_Test_ parameters, which is due to the increased PSII stability after exposure to supra-optimal temperatures. To test these hypotheses, two *C. canephora* clones known to differentiate in their sensitivity to drought were subjected to two cycles of water deficit followed by rehydration. Finally, heat shock using a water bath was simulated in both clones and the ChlF emission from the samples was monitored.

## 2. Results

**Figure 1:**
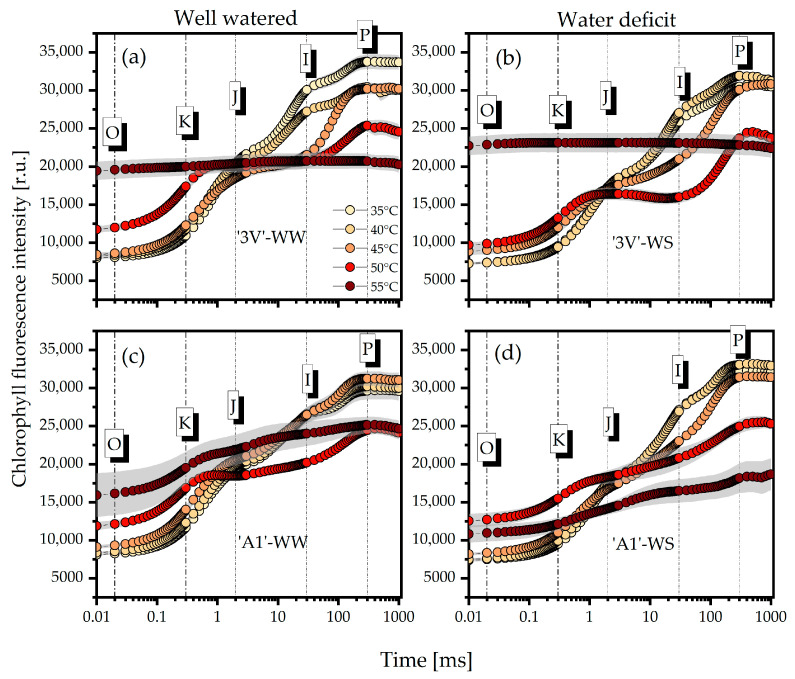
Chlorophyll *a* fluorescence induction curves, as a function of time, in logarithmic scale, measured on leaf discs subjected to heat treatments in a water bath, at different temperatures. Samples were collected from *Coffea canephora* ‘3V’ and ‘A1’ clones grown under well-irrigated conditions (WW) and under two cycles of recurrent water deficit (WS). The figure contains data related to the following treatments: (**a**) ‘3V’-WW; (**b**) ‘3V’-WS; (**c**) ‘A1’-WW and (**d**) ‘A1’-WS—all incubated for 15 min at 35 °C, 40 °C, 45 °C, 50 °C and 55 °C. Temperature intensity was indicated by color intensity: lighter colors represent lower temperatures and darker colors higher temperatures (details in figure (**a**)). Each symbol indicates the mean of n = 5. Within each figure, O, K, J, I and P represent the ChlF emission curve steps at times 20 µs, 300 µs, 2 ms, 30 ms and 300 ms, respectively.

The analysis of polyphasic OJIP transient curves of the ‘3V’ and ‘A1’ clones under irrigated (WW) and post-water deficit (WS) conditions showed that high temperatures caused considerable changes in the curve steps starting from 45 °C (Figure 1). At 55 °C, the ‘3V’-WW (Figure 1a) and ‘3V’-WS (Figure 1b) treatments exhibited a complete loss of the characteristic rise in the fluorescence induction curve between the O and P steps. Although a rise in the O step was generally observed under elevated temperatures, the ‘3V’-WS (Figure 1b) and ‘A1’-WS (Figure 1d) treatments showed lower rises compared to the control (35 °C) at 50 and 55 °C, respectively. The appearance of the K step, which became evident starting from 45 °C, was the most pronounced at 50 °C in the ‘3V’-WW (Figure 1a) and ‘A1’-WW (Figure 1c) treatments. Only slight variations were observed in the J step with increasing temperature, which became evident at 55 °C for the ‘3V’-WS (Figure 1b) and ‘A1’-WS (Figure 1d) treatments. In general, the amplitude of the I step decreased with increasing temperature, particularly in the ‘3V’-WW (Figure 1a) and ‘3V’-WS (Figure 1b) treatments starting from 45 °C. The decline in the P step amplitude induced by temperature rise was especially pronounced in the ‘3V’-WW (Figure 1a) and ‘A1’-WS (Figure 1d) treatments. The normalizations of each stage between O and P are presented in order to highlight the differences among the treatments.

**Figure 2:**
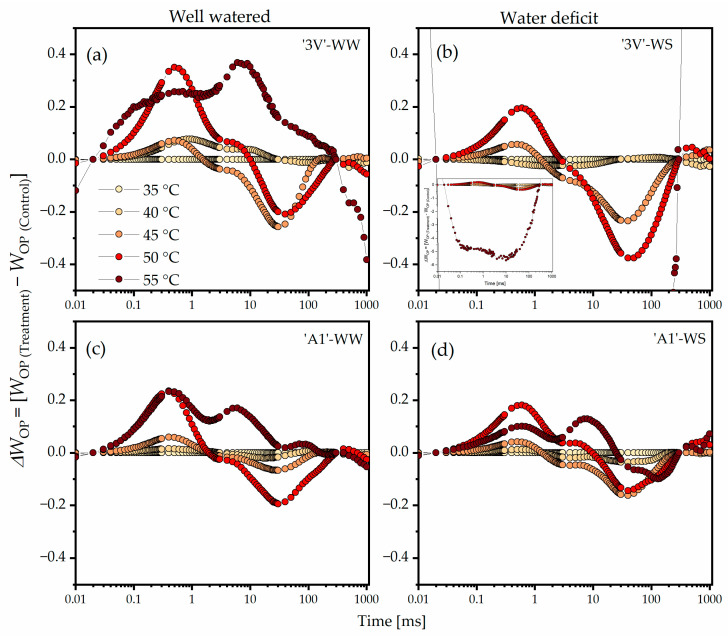
Differential analysis of OJIP kinetics (*ΔW*_OP_), obtained by normalization of relative Chl *a* fluorescence kinetics between the O and P steps (*W*_OP_) in the chlorophyll *a* fluorescence induction curves measured on leaf discs subjected to heat treatments in a water bath, at different temperatures. Leaf discs were collected from *Coffea canephora* ‘3V’ and ‘A1’ clones grown under well-irrigated conditions (WW) and under two cycles of recurrent water deficit (WS). The figure contains data related to the following treatments: (**a**) ‘3V’-WW; (**b**) ‘3V’-WS; (**c**) ‘A1’-WW and (**d**) ‘A1’-WS—all incubated for 15 min at 35 °C, 40 °C, 45 °C, 50 °C and 55 °C. Temperature intensity was indicated by color intensity: lighter colors represent lower temperatures and darker colors higher temperatures (details in figure (**a**)). Each symbol indicates the mean of n = 5. The ordinate on the graphs shows values of *W*_OP_ = (*F*_t_ − *F*_0_)/(*F*_P_ − *F*_0_) and *ΔW*_OP_ = (*W*_OP(Temperature)_ − *W*_OP(35°C)_).

Considering the double normalization of *W*_OP_ and *ΔW*_OP_ (Figure 2) between the O step (*F*_20µs_) and the P step (*F*_300ms_) of the typical polyphasic Chl *a* fluorescence transient curve, trends toward increased positive amplitude at the K step and increased negative amplitude at the I step were evident starting from 45 °C, particularly at 50 °C and 55 °C. Clear changes in *ΔW*_OP_ due to elevated temperatures were expressed in the following order: ‘3V’-WS (Figure 2b) > ‘3V’-WW (Figure 2a) > ‘A1’-WW (Figure 2c) > ‘A1’-WS (Figure 2d). A complete loss of photosynthetic function from the O step was observed in ‘3V’-WS at 55 °C (Figure 2b), indicated by the high negative amplitude throughout the fluorescence kinetics.

**Figure 3:**
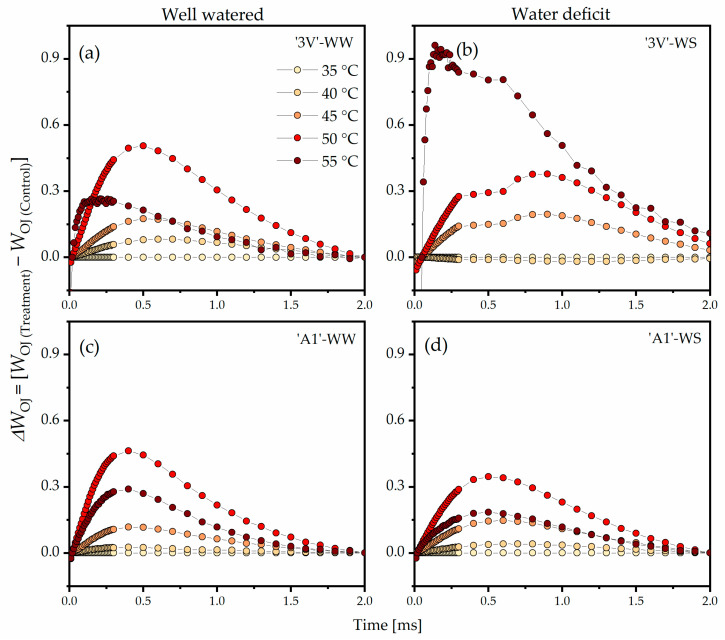
Differential analysis of the OJIP kinetics (*ΔW*_OJ_) of Chl *a* fluorescence transient, obtained by normalizing their relative values between the O and J steps (*W*_OJ_) in the chlorophyll *a* fluorescence induction curves measured on leaf discs subjected to heat treatments in a water bath, at different temperatures. Leaf discs were collected from *Coffea canephora* ‘3V’ and ‘A1’ clones grown under well-irrigated conditions (WW) and under two cycles of recurrent water deficit (WS). The figure contains data related to the following treatments: (**a**) ‘3V’-WW; (**b**) ‘3V’-WS; (**c**) ‘A1’-WW and (**d**) ‘A1’-WS—all incubated for 15 min at 35 °C, 40 °C, 45 °C, 50 °C and 55 °C. Temperature intensity was indicated by color intensity: lighter colors represent lower temperatures and darker colors higher temperatures (details in figure (**a**)). Each symbol indicates the mean of n = 5. Further, *W*_OJ_ = (*F*_t_ − *F*_O_)/(*F*_J_ − *F*_O_) and *ΔW*_OJ_ = (*W*_OJ(Temperature)_ − *W*_OJ(35°C)_) were normalized.

The double normalization of *W*_OJ_ and *ΔW*_OJ_ (Figure 3) between the O step (*F*_O_) and the J step (*F*_J_) revealed the K-band, which indicated that the rise in temperatures from 35 °C to 45 °C, 50 °C, or 55 °C led to positive amplitude increase of Δ*W*_OJ_ in all treatments. In the ‘3V’-WW, this increase began at 40 °C and was the highest at 50 °C (Figure 3a). In ‘3V’-WS, only a slight increase of Δ*W*_OJ_ was observed at 40 °C when compared to 35 °C (Figure 3b). The Δ*W*_OJ_ rise in ‘3V’-WS was lower when compared to ‘3V’-WW at 50 °C, while at 55 °C, a significant rise in the amplitude of *ΔW*_OJ_ was observed, indicating severe damage of the photosynthetic apparatus. In ‘A1’-WW (Figure 3c) and in ‘A1’-WS (Figure 3d), an increase in amplitude of *ΔW*_OJ_ due to temperature increase was observed, although it was less pronounced than in ‘3V’-WW and ‘3V’-WS. Additionally, the peaks of *ΔW*_OJ_ in ‘A1’-WS at 50 °C and 55 °C were relatively smaller compared to those in ‘A1’-WW.

**Figure 4:**
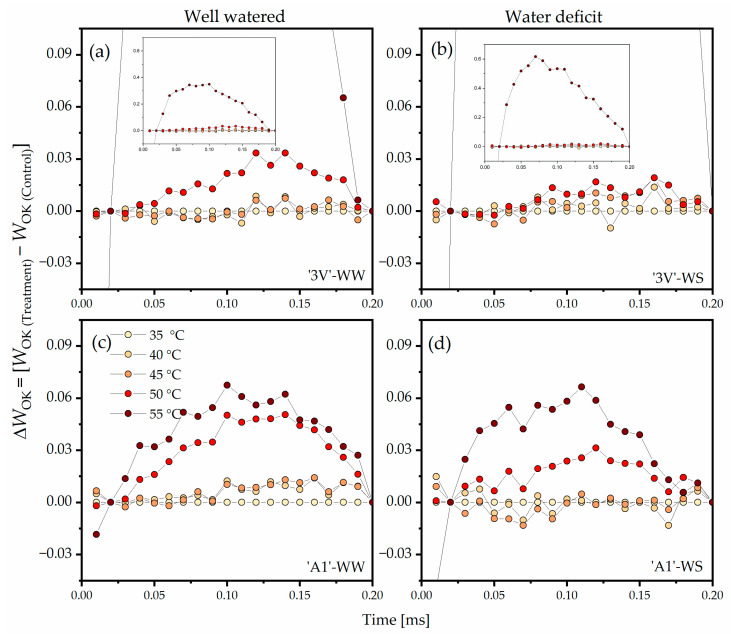
Differential analysis of the OJIP kinetics (*ΔW*_OK_), obtained by normalizing the relative chlorophyll *a* fluorescence kinetics between the O and K steps (*W*_OK_) in transient induction curves measured on leaf discs subjected to heat treatments in a water bath, at different temperatures. Leaf discs were collected from *Coffea canephora* ‘3V’ and ‘A1’ clones grown under well-irrigated conditions (WW) and under two cycles of recurrent water deficit (WS). The figure contains data related to the following treatments: (**a**) ‘3V’-WW; (**b**) ‘3V’-WS; (**c**) ‘A1’-WW and (**d**) ‘A1’-WS—all incubated for 15 min at 35 °C, 40 °C, 45 °C, 50 °C and 55 °C. Temperature intensity was indicated by color intensity: lighter colors represent lower temperatures and darker colors higher temperatures (details in figure (**c**)). Each symbol indicates the mean of n = 5. The data were normalized as: *W*_OK_ = (*F*_t_ − *F*_O_)/(*F*_K_ − *F*_O)_ and *ΔW*_OK_ = (*W*_OK(Temperature)_ − *W*_OK(35°C)_).

The double normalization of *W*_OK_ and *ΔW*_OK_ between the O step (*F*_O_) and the K step (*F*_K_) revealed the L-band (Figure 4). In ‘3V’-WW, an increase of *ΔW*_OK_ amplitude at 50 °C and 55 °C was noted, indicating extreme damage at the highest temperatures (Figure 4a). In ‘3V’-WS, the peak of *ΔW*_OK_ at 50 °C was much lower compared to that in ‘3V’-WW at the same temperature (Figure 4b), but the high amplitude persisted at 55 °C in all treatments. ‘A1’-WW (Figure 4c) and ‘A1’-WS (Figure 4d) showed higher *ΔW*_OK_ stability, with relatively lower peak amplitudes in ‘A1’-WS compared to ‘A1’-WW at temperatures below 55 °C.

**Figure 5:**
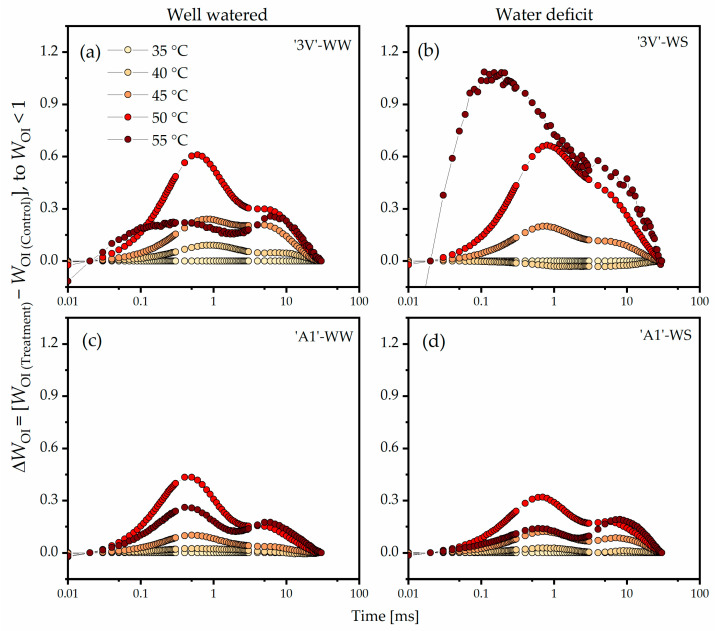
Differential analysis of the OJIP kinetics (*ΔW*_OI_, *W*_OI_ < 1) of the Chl *a* fluorescence transient curve, obtained by normalizing the relative fluorescence kinetics between the O and I steps (*W*_OI_ < 1) in chlorophyll *a* fluorescence induction curves measured on leaf discs subjected to heat treatments in a water bath, at different temperatures. Leaf discs were collected from *Coffea canephora* ‘3V’ and ‘A1’ clones grown under well-irrigated conditions (WW) and under two cycles of recurrent water deficit (WS). The figure contains data related to the following treatments: (**a**) ‘3V’-WW; (**b**) ‘3V’-WS; (**c**) ‘A1’-WW and (**d**) ‘A1’-WS—all incubated for 15 min at 35 °C, 40 °C, 45 °C, 50 °C and 55 °C. Temperature intensity was indicated by color intensity: lighter colors represent lower temperatures and darker colors higher temperatures (details in figure (**a**)). Each symbol indicates the mean of n = 5. The data were normalized as: *W*_OI_ = (*F*_t_ −*F*_O_)/(*F*_I_ − *F*_O_) to *W*_OI_ < 1 and *ΔW*_OI_ = (*W*_OI(Temperature)_ − *W*_OI(35°C)_).

Considering the double normalization of *W*_OI_ and *ΔW*_OI_ (*W*_OI_ < 1) between the O step (*F*_O_) and the I step (*F*_I_), the amplitude of the curve in ‘3V’-WW at 40 °C was slightly higher than at 35 °C, with the greatest amplitude observed at 50 °C (Figure 5a). The highest amplitude of *ΔW*_OK_ in ‘3V’-WS was observed at 55 °C, with significant variations (Figure 5b). The ‘A1’ clone (Figure 5c,d) exhibited reduced *ΔW*_OK_ curve amplitudes starting from 45 °C, especially under WS, when compared to the ‘3V’ clone (Figure 5a,b).

**Figure 6:**
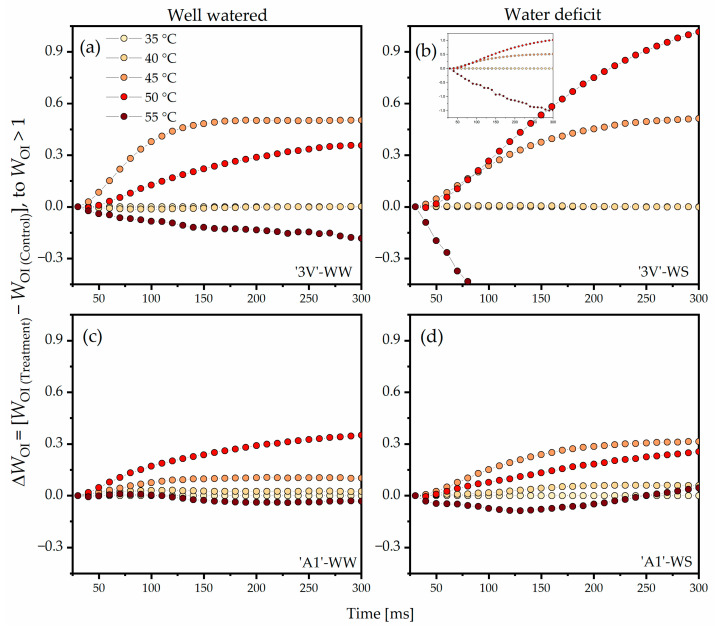
Differential analysis of the OJIP kinetics (*ΔW*_OI_, *W*_OI_ > 1), obtained by normalizing the relative Chl *a* fluorescence kinetics between the O and I steps (*W*_OI_ > 1) in the induction curves measured on leaf discs subjected to heat treatments in a water bath, at different temperatures. Leaf discs were collected from *Coffea canephora* ‘3V’ and ‘A1’ clones grown under well-irrigated conditions (WW) and under two cycles of recurrent water deficit (WS). The figure contains data related to the following treatments: (**a**) ‘3V’-WW; (**b**) ‘3V’-WS; (**c**) ‘A1’-WW and (**d**) ‘A1’-WS—all incubated for 15 min at 35 °C, 40 °C, 45 °C, 50 °C and 55 °C. Temperature intensity was indicated by color intensity: lighter colors represent lower temperatures and darker colors higher temperatures (details in figure (**a**)). Each symbol indicates the mean of n = 5. The data were normalized: *W*_OI_ = (*F*_t_ − *F*_O_)/(*F*_I_ − *F*_O_) to *W*_OI_ > 1 and *ΔW*_OI_ = (*W*_OI(Temperature)_ − *W*_OI(35°C)_).

The highest positive amplitudes of the double normalization of *W*_OI_ and *ΔW*_OI_ (*W*_OI_ > 1) in ‘3V’-WW were observed at 50 °C and 45 °C, while a negative amplitude was noted at 55 °C when compared to the control (35 °C) (Figure 6a). In ‘3V’-WS, the highest amplitudes of *ΔW*_OI_ were observed at 45 °C and 50 °C, while a negative amplitude was also noted at 55 °C when compared to the control (35 °C) (Figure 6b). The *ΔW*_OI_ amplitudes observed in ‘3V’-WS (Figure 6b) were greater than in ‘3V’-WW (Figure 6a). The highest *ΔW*_OI_ amplitudes in ‘A1’-WW were noted at 45 °C and 50 °C (Figure 6c), while in ‘A1’-WS, the highest *ΔW*_OI_ amplitudes were observed at 50 °C and 45 °C (Figure 6d). At 55 °C, the negative amplitudes of *ΔW*_OI_ in ‘A1’-WW and ‘A1’-WS were lower than in ‘3V’-WW and ‘3V’-WS.

**Figure 7:**
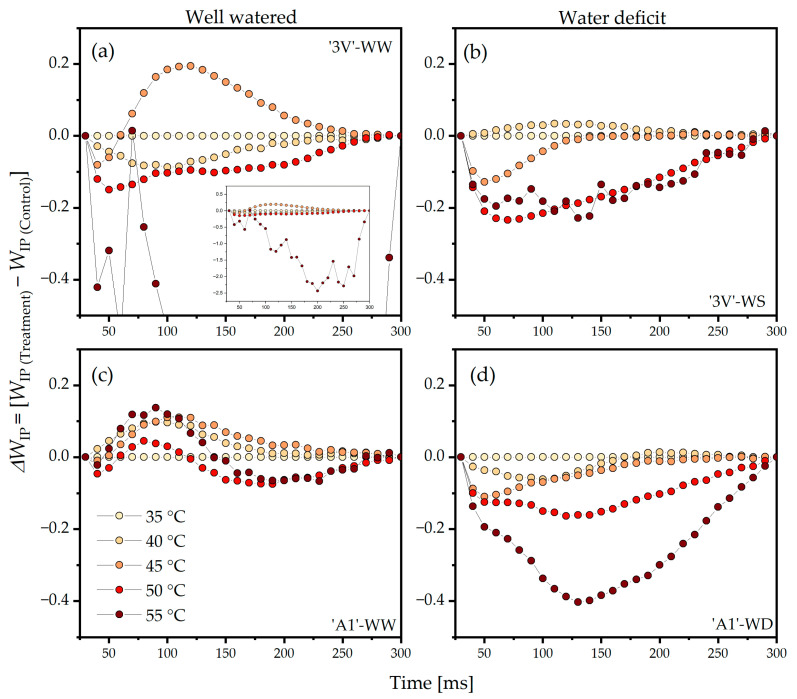
Differential analysis of the OJIP kinetics (*ΔW*_IP_), obtained by normalizing the relative Chl *a* fluorescence kinetics between the I and P steps (*W*_IP_) in the transient induction curves measured on leaf discs subjected to heat treatments in a water bath, at different temperatures. Leaf discs were collected from *Coffea canephora* ‘3V’ and ‘A1’ clones grown under well-irrigated conditions (WW) and under two cycles of recurrent water deficit (WS). The figure contains data related to the following treatments: (**a**) ‘3V’-WW; (**b**) ‘3V’-WS; (**c**) ‘A1’-WW and (**d**) ‘A1’-WS—all incubated for 15 min at 35 °C, 40 °C, 45 °C, 50 °C and 55 °C. Temperature intensity was indicated by color intensity: lighter colors represent lower temperatures and darker colors higher temperatures (details in figure (**c**)). Each symbol indicates the mean of n = 5. The data were normalized as: *W*_IP_ = (*F*_t_ − *F*_O_)/(*F*_I_ − *F*_P_) and *ΔW*_IP_ = (*W*_IP(Temperature)_ − *W*_IP(35°C)_).

The analysis of the double normalization of *W*_IP_ and *ΔW*_IP_ between the step I (*F*_I_) and the step P (*F*_M_) revealed that the greatest positive amplitude in ‘3V’-WW was observed at 45 °C, while the greatest negative amplitudes were expressed at 40 °C, 50 °C, and 55 °C (Figure 7a). The amplitude of *ΔW*_IP_ was slightly positive in ‘3V’-WS at 40 °C, while negative amplitudes were observed at 45 °C, 50 °C, and 55 °C (Figure 7b). The *ΔW*_IP_ amplitude in ‘3V’-WS at 55 °C was lower when compared to ‘3V’-WW (Figure 7a). All curves of *ΔW*_IP_ in ‘A1’-WW showed positive amplitudes, with no significant variations among the temperatures (Figure 7c). Interestingly, the *ΔW*_IP_ curves in ‘A1’-WS showed negative amplitudes at 40 °C, 45 °C, 50 °C, and 55 °C (Figure 7d).

Concerning energy flux ratios or the quantum yields, the effect of interaction between treatments and temperature was significant for φ_Po_, φ_Eo_, φ_Do_, and φ_Ro_, but not for Ψ_Eo_ and δ_Ro_ (Table S1). The Ψ_Eo_ showed a significant difference among the treatments and temperatures, while the δ_Ro_ showed significant differences only among applied temperatures.

**Figure 8:**
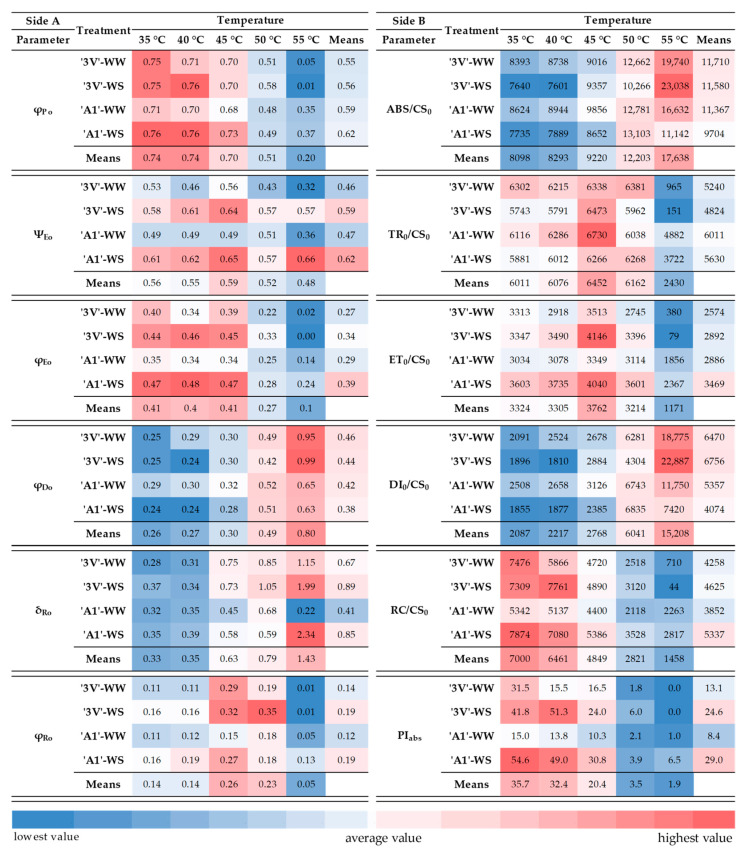
Heatmap for (Side A) quantum efficiency or the energy flux ratio parameters and (Side B) parameters of phenomenological energy flux and photosynthetic indices derived from the analysis of OJIP transients using JIP_Test_, measured in leaf discs subjected to heat treatments at different temperatures (35 °C, 40 °C, 45 °C, 50 °C, and 55 °C) and incubated for 15 min in a water bath. Discs were collected from *Coffea canephora* ‘3V’ and ‘A1’ clones grown under well-irrigated conditions (WW) and under two cycles of recurrent water deficit (WS), resulting in the following treatments: ‘3V’-WW, ‘3V’-WS, ‘A1’-WW, and ‘A1’-WS. Note: φ_Po_ represents the maximum quantum yield of primary PSII photochemistry; Ψ_Eo_, the probability with which a PSII trapped electron is transferred from Q_A_ to Q_B_; φ_Eo_, the quantum yield of electron transport flux from Q_A_ to Q_B_; φ_Do_, the quantum yield of energy dissipation; δ_Ro_, the probability with which an electron from Q_B_ is transferred until it reaches the PSI electron acceptor(s); φ_Ro_, the quantum yield of electron transport flux until it reaches the PSI electron acceptors; ABS/CS_0_, absorbed energy; TR_0_/CS_0_, energy flux trapped by PSII reaction centers; ET_0_/CS_0_, electron transfer through PSII; DI_0_/CS_0_, thermal dissipation of energy in PSII; RC/CS_0_, density of reaction centers capable of Q_A_ reduction, described per unit of leaf cross-section (CS); PI_abs_, PSII performance index normalized by absorbed energy. Blue colors indicate low values, and red colors indicate high values for each parameter.

The φ_Po_ values did not vary at temperatures between 35 °C and 45 °C, despite the trend of reduction (Figure 8, Side A; Table S2). Significant decreases by 32%, 23%, 32%, and 36% in φ_Po_ were observed at 50 °C compared to 35 °C in ‘3V’-WW, ‘3V’-WS, ‘A1’-WW, and ‘A1’-WS, respectively. Significant differences in φ_Po_ among treatments were observed at 55 °C, where ‘A1’-WW and ‘A1’-WS were similar and statistically higher than ‘3V’-WW and ‘3V’-WS, which additionally did not differ from each other. At 55 °C, φ_Po_ decreased by 93% and 98% in ‘3V’-WW and ‘3V’-WS, respectively, and by 51% in ‘A1’-WW and ‘A1’-WS, indicating that the ‘A1’ clone has greater heat tolerance than ‘3V’.

A significant decrease in Ψ_Eo_ was observed at 55 °C in all treatments (Figure 8, Side A; Table S2). Ψ_Eo_ mean values for overall temperatures indicated that the treatments ‘3V’-WS and ‘A1’-WS were approximately 25% higher when compared to ‘3V’-WW and ‘A1’-WW.

The tendencies of higher mean values of φ_Eo_ in ‘3V’-WS and ‘A1’-WS were expressed at temperatures ranging from 35 °C to 45 °C when compared to their counterparts, ‘3V’-WW and ‘A1’-WW, respectively (Figure 8, Side A; Table S2). At 50 °C, the φ_Eo_ was higher only in ‘3V’-WS than in ‘3V’-WW. The lowest overall values of φ_Eo_ were observed in all treatments at 50 °C and 55 °C, with emphasis in ‘3V’ at 55 °C. At 55 °C, the φ_Eo_ was reduced by 95%, 99%, 60%, and 49% in ‘3V’-WW, ‘3V’-WS, ‘A1’-WW, and ‘A1’-WS, respectively, compared to control (35 °C).

A response of φ_Do_ was inversely proportional to that observed in φ_Po_ (Figure 8, Side A; Table S2). No difference in φ_Do_ was observed between 35 °C and 45 °C among treatments. The highest mean values of φ_Do_ were observed at 50 °C and 55 °C, with emphasis on ‘3V’ at 55 °C. The φ_Do_ showed different values among different treatments only at 55 °C, where φ_Do_ in ‘A1’-WW and ‘A1’-WS was lower than in ‘3V’-WW and ‘3V’-WS.

A considerable increase in δ_Ro_ was observed starting from 45 °C, with the highest value observed at 55 °C (Figure 8, Side A; Table S2). Higher φ_Ro_ means were observed at 45 °C and 50 °C, and for the treatments ‘3V’-WS and ‘A1’-WS, when compared to their counterparts ‘3V’-WW and ‘A1’-WW, respectively. In addition, at 50 °C, ‘3V’-WS showed higher δ_Ro_ compared to other treatments. At 55 °C, the φ_Ro_ of the clone ‘3V’ was lower than that of ‘A1’.

All the phenomenological energy flow parameters per leaf cross-section and performance indices were significantly impacted by the interaction between treatment and temperature (Table S1). The highest temperatures showed the highest mean values of ABS/CS_0_, especially at 55 °C, suggesting the increased energy absorption. At this temperature, a difference among the treatments in ABS/CS_0_ was observed, resulting in the following order: ‘A1’-WS < ‘A1’-WW < ‘3V’-WW < ‘3V’-WS.

The TR_0_/CS_0_ differed among treatments only at 55 °C, where ‘A1’-WW and ‘A1’-WS preserved significantly higher values than those of ‘3V’-WW and ‘3V’-WS (Figure 8, Side B; Table S3). This parameter suffered reductions of 84%, 97%, 20%, and 36% at 55 °C compared to control (35 °C) for ‘3V’-WW, ‘3V’-WS, ‘A1’-WW and ‘A1’-WS, respectively. The exception was ‘A1’-WW treatment, where the highest TR_0_/CS_0_ value was observed at 45 °C. These results showed that even under high temperatures, the RCs of PSII of genotype ‘A1’ were able to capture the energy absorbed by light-harvesting (antenna) complexes of PSII (LHCII).

The ET/CS_0_ decreased by 89%, 97%, 39%, and 34% at 55 °C compared to 35 °C in ‘3V’-WW, ‘3V’-WS, ‘A1’-WW, and ‘A1’-WS, respectively (Figure 8, Side B; Table S3). Furthermore, the general mean ET/CS_0_ values for ‘A1’-WW and ‘A1’-WS remained significantly higher than those for ‘3V’-WW and ‘3V’-WS. This demonstrated that the ‘A1’ genotype maintained active electron transport even at high temperatures.

The highest general mean values of DI_0_/CS_0_ were observed at 55 °C for most of the treatments, with the exception of A1’-WS, which showed elevated DI_0_/CS_0_ values at both 50 °C and 55 °C (Figure 8, Side B; Table S3). The treatments differed from each other in DI_0_/CS_0_ at 55 °C, resulting in the following order: ‘A1’-WS < ‘A1’-WW < ‘3V’-WW < ‘3V’-WS. This indicated that clone ‘A1’ was more efficient than clone ‘3V’ in dissipating energy excess under high temperatures, and that this efficiency was further boosted by drought priming.

Differences in RC/CS_0_ were observed starting from 45 °C in all treatments (Figure 8, Side B; Table S3). In both ‘3V’-WW and ‘3V’-WS, the RC/CS_0_ decreased by 35%, 62%, and 95% at 45 °C, 50 °C, and 55 °C, respectively. The RC/CS_0_ was reduced by 60% in ‘A1’-WW at 50 °C and 55 °C. In ‘A1’-WS, this parameter suffered a reduction of 32% at 45 °C, and about 60% at 50 °C and 55 °C. The RC/CS_0_ in ‘A1’-WS was higher than in ‘A1’-WW at 50 °C, with no difference among the other treatments. This parameter had significantly higher values in ‘A1’-WW and ‘A1’-WS than in ‘3V’-WW and ‘3V’-WS at 55 °C.

Temperatures above 40 °C caused reductions in PI_abs_ in all treatments, except in ‘A1’-WW (Figure 8, Side B; Table S3). PI_abs_ was reduced by about 50% at 40 °C and 45 °C, and by 97% at 50 °C and 55 °C in ‘3V’-WW. This parameter was reduced by 42%, 85%, and 99% at 45 °C, 50 °C, and 55 °C, respectively, in ‘3V’-WS. PI_abs_ was reduced by 44%, 93%, and 88% at 45 °C, 50 °C, and 55 °C, respectively, in ‘A1’-WS. Overall, the average PI_abs_ values for ‘3V’-WS and ‘A1’-WS were 1.5-fold higher compared to their counterparts, ‘3V’-WW and ‘A1’-WW.

**Figure 9:**
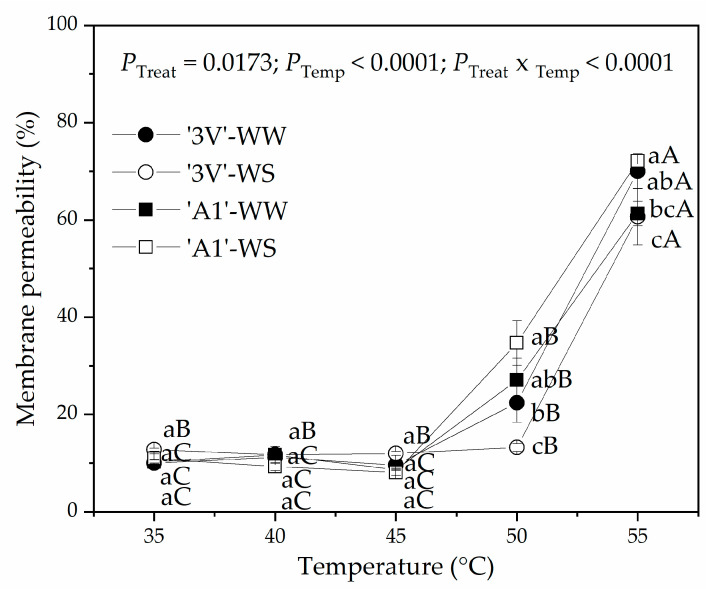
Membrane permeability (%) determined in leaf discs subjected to heat treatments in a water bath at different temperatures. Leaf discs were collected from *Coffea canephora* ‘3V’ and ‘A1’ clones grown under well-irrigated conditions (WW) and under two cycles of recurrent water deficit (WS). The figure contains data related to the following treatments (_Trait_): ‘3V’-WW, ‘3V’-WS, ‘A1’-WW and ‘A1’-WS—all incubated for 15 min at temperatures (_Temp_) of 35 °C, 40 °C, 45 °C, 50 °C and 55 °C. Each symbol represents a mean value, and the vertical bars represent the standard error (SE) for n = 5. ANOVA *p*-values < 0.05 were considered significant. Lowercase letters compare means of treatments at each temperature, while uppercase letters compare means of temperatures for each treatment according to the Tukey test at 95% confidence.

The effect of interaction between treatments and temperatures was expressed on membrane permeability (Figure 9). No difference among the treatments was observed up to 45 °C. The increase in membrane permeability was observed at 50 °C and 55 °C. A lower increase in membrane permeability was noted in ‘3V’-WS compared with other treatments at 50 °C. The membrane permeability was higher in ‘A1’-WS compared with ‘3V’-WS only at 55 °C.

## 3. Discussion

**Figure 10:**
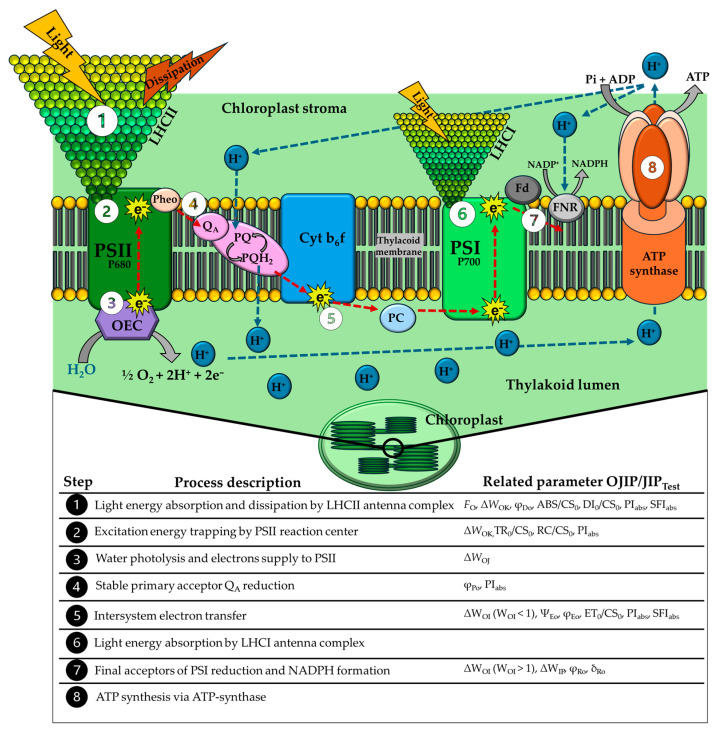
Schematic presentation of the photosynthetic apparatus in the thylakoid membranes of a chloroplast indicating the OJIP/JIP_Test_ parameters related to each energy-transfer step in the electron-transport chain (ETC). LHCII—light-harvesting complex of photosystem II; PSII—photosystem II reaction center (P680); OEC—oxygen-evolving complex; e^-^—electron; Pheo—pheophytin; Q_A_—quinone A molecule; PQ—pool of plastoquinone; PQH_2_—plastohydrolquinol; Cyt b6/f—cytochrome b6/f; PC—plastocyanin; LHCI—light-harvesting complex of photosystem I; PSI—photosystem I reaction center (P700); Fd—ferredoxin; FNR—ferredoxin-NADP^+^ reductase; NADP^+^—oxidized nicotinamide adenine dinucleotide phosphate; NADPH—reduced nicotinamide adenine dinucleotide phosphate; Pi—inorganic phosphate; ADP—adenosine diphosphate; ATP—adenosine triphosphate; H^+^—hydrogen proton. Red dotted arrows indicate the electron flow in the ETC, blue dotted arrows indicate the H^+^ flow around the ETC, and grey arrows indicate molecule synthesis.

This study investigated the effects of two recurrent water deficits on the induction of heat stress tolerance in *C. canephora* clones ‘3V’ and ‘A1’, grown in a greenhouse and exposed to mean and maximum temperatures of up to 30 °C and 41 °C, respectively. The analysis here focused on the changes in the OJIP kinetics of chlorophyll *a* fluorescence (ChlF) emission curves and utilized quantitative assessments based on the JIP_Test_ parameters [44]. Previous findings indicated that clone ‘3V’ exhibited drought tolerance following two cycles of water deficit, being less influenced in leaf net CO_2_ assimilation rate, effective quantum yield in PSII photochemistry, photochemical quenching, linear electron transport rate, and photochemical reflectance index [6], which was supported by some morphological traits—particularly the root growth [45]. In contrast, clone ‘A1’ displayed greater sensitivity in the above-mentioned physiological parameters [6], but demonstrated a more conservative water-use strategy for growth [45]. Recent studies in *Coffea* species have demonstrated a complex cross-interaction between drought and heat stress events, generally in a genotype-dependent manner [5,17]. They indicate that, in some cases, the simultaneous or successive occurrence of these stress events can trigger protective responses in PSII and in the photosynthetic apparatus. Such protective responses are commonly associated with the activity of photoprotective compounds, such as zeaxanthin, and antioxidant enzymes, as well as with the synthesis of non-structural carbohydrates, alterations in fatty acid profiles, modulation of gene expression, and the activity of heat shock proteins [46]. To facilitate the discussion interpretation, we show the schematic of the photosynthetic apparatus indicating the OJIP/JIP_Test_ parameters related to each energy-transfer step in the ETC (Figure 10).

The *W*_OP_ normalization denotes the relative variable Chl *a* fluorescence at each t point between the O and P steps, also called *V*_t_ [36]. The *ΔW*_OP_ normalization value indicated the subtraction of the variable fluorescence (*V*) of the control sample (here 35 °C) from the treated samples. This normalization reveals much more information usually hidden in the Chl *a* fluorescence rise kinetics [38]. A more pronounced peak at the J step and a less pronounced peak at the I step indicate a slowdown in the electron transport between the J and I [36]. Our results indicated a greater amplitude of variation in *ΔW*_OP_ for clone ‘3V’ than for ‘A1’, especially after water deficit cycles (Figure 2). This may indicate a decrease in electron transfer along the ETC, revealing the greater sensitivity of ‘3V’ to increased temperatures, which may be enhanced by previously experienced water deficit.

In plants, increased cellular membrane fluidity is the main effect that triggers damage to the photosynthetic apparatus and, consequently, carbon fixation [47,48,49]. We observed an increase in membrane permeability at 50 °C and 55 °C, indicating an increase in membrane fluidity (Figure 9), but PSII has been reported to be much more sensitive to heat than membrane stability [16]. Changes in fatty acid composition and the activity of heat shock proteins are associated with enhanced thermostability in plants growing in warm environments or undergoing long-term acclimation [50]. However, in our study, short-term heat shock was simulated, under which no adjustments in membrane thermostability were observed.

The photochemical reactions, especially electron transfer, occur in the thylakoid membranes, whose lipid composition makes them sensitive to temperature increases [11,51,52]. Supra-optimal temperatures cause the dissociation of pigment-protein complexes, such as LHCII and PSII, and other components of the ETC, such as OEC, and Cyt b6/f [53,54]. This compromises electron flow, the generation of ATP and the reducing power NADPH [54]. In ChlF, the dissociation between LHCII and PSII was interpreted from the increase in *F*_0_ or the O step in the OJIP transient (Figure 10, step 1), associated with increased ChlF emission by the antenna complexes and reduced excitation energy transfer to PSII [33,41,55]. Our results showed an increase in *F*_0_ or the O level (Figure 1) at temperatures above 45 °C; however, at 55 °C, clone ‘3V’ was shown to be more sensitive, due to a significant increase in *F*_0_, compared to clone ‘A1’. Additionally, lower values of *F*_0_ were observed in the ‘3V’-WS treatments at 50 °C and in ‘A1’-WS at 55 °C. These results were supported by those observed in the double normalization *W*_OK_ and *ΔW*_OK_ (Figure 4), where the positive amplitude of the kinetics of *ΔW*_OK_ was related to the loss of connectivity among the structural PSII components (Figure 10, steps 1 and 2), i.e., LHC and antenna core coupled to RCs, and the RCs themselves [36]. This amplitude is evidenced at high temperatures [56,57] and can be quantified by the increases observed in *W*_L_ (Table S4). Greater amplitudes in *ΔW*_OK_ were observed at 55 °C in ‘3V’-WW and ‘3V’-WS, while the lowest amplitudes were at 50 °C in ‘3V’-WS and ‘A1’-WS compared to their counterparts, ‘3V’-WW and ‘A1’-WW (Figure 4). Our results may indicate a possible acclimatization of the photosynthetic apparatus in both clones, triggered by the recurring water deficit under moderate to high temperatures in the growing environment.

The thermal stress caused the appearance of an additional K step between the O and J points, also known as the K-band (Figure 1), which becomes visible at 200–300 µs [41,58] and can be quantified by the increase in the *W*_K_ parameter (Table S4). At the highest observed temperatures (50 and 55 °C), the limitations on the electron donor side (OEC, K-band) and the energy dysconnectivity between the PSII components (L-band) occurred. At such a state, alternative electron donors briefly reduced P680^+^ and Q_A_, but due to inability to maintain electron flow towards Q_B_, the P680^+^ accumulation was induced and fluorescence decreased from 300 µs onwards, making the K-point apparent [57]. The appearance of this K step is primarily related to a limitation in the efficiency of the electron donor side of the OEC [36,37] and can be quantified by analyzing the *F*_K_/*F*_J_ parameter (Table S4; Figure 10, step 3), whose increases are proportional to the damage caused by high temperatures [59,60]. Furthermore, the reduction of the parameter related to OEC (Table S4; Figure 10, step 3) indicates the inactivation of OEC [38]. The double normalization *W*_OJ_ and *ΔW*_OJ_ (Figure 3) revealed the appearance of the K-band, and its positive amplitude was associated with greater inactivation of the OEC (Figure 10, step 3), which limits the supply of electrons to the ETC [37]. Indeed, temperatures above 35 °C have been shown to result in a significant reduction in O_2_ release in *Carica papaya* L. [61]. However, in *Coffea* spp., no significant impact was observed on thermotolerance for O_2_ release up to 42 °C [5]. Our results indicated a greater amplitude of the *ΔW*_OJ_ curve at 50 °C for the treatments ‘3V’-WW, ‘A1’-WW and ‘A1’-WS, except for ‘3V’-WS, where the greatest amplitude was observed at 55 °C. Furthermore, we did not observe significant differences among WW and WS treatments for this variable (Figure 3), indicating that water deficit did not induce greater stability of the OEC.

The inactivation of the OEC leads to the emergence of alternative electron donors, such as proline, which promotes the rapid reduction of Q_A_ and an increase in Chl *a* fluorescence at 300 µs [62]. Alternative electron donors are not able to maintain a continuous flow of electrons beyond Q_B_, leading to the accumulation of P680^+^ and consequent reduction in fluorescence yield between the J and P steps (*F*_M_) [63]. When examining the double normalized values of *W*_OI_ and *ΔW*_OI_, the positive amplitude for *W*_OI_ < 1 (Figure 5) could be related to the blockage of electrons from PSII to plastoquinone (Figure 10, step 5) [38]. Our results showed a significant increase in amplitude of *ΔW*_OI_ < 1, starting from 45 °C in ‘3V’-WW and ‘3V’-WS, while the amplitudes for ‘A1’-WW and ‘A1’-WS were lower. Additionally, the lower amplitudes of ‘A1’-WS compared to ‘A1’-WW may indicate the clone’s ability to maintain electron flow beyond Q_A_ even under WS. The values of Ψ_Eo_, φ_Eo_ (Figure 8, Side A; Table S2), and ET_0_/CS_0_ (Figure 8, Side B; Table S3), parameters related to electron transport along the ETC (Figure 10, step 5), did not show clear differences among treatments. However, for these parameters, the mean values for ‘A1’-WS might indicate that this treatment performed better here than ‘3V’-WW, ‘3V’-WS and ‘A1’-WW in maintaining electron transport beyond Q_A_.

The double normalized value of *W*_OI_ and *ΔW*_OI_ to *W*_OI_ > 1 reflects the size of the final PSI electron acceptor pool due to the electron flow driven by PSI beyond PQH_2_ [37,38]. Therefore, the positive amplitude of the *ΔW*_OI_ curve (*W*_OI_ > 1) in *C. canephora* was related to a larger pool of active final PSI acceptors (Figure 10, step 7). Temperatures of 45 °C and 50 °C induced increased activity of final PSI acceptors in all treatments, with the effect being most evident in ‘3V’-WW and ‘3V’-WS (Figure 6). Indeed, higher temperatures are associated with increased PSI activity, stimulating intersystem electron flow [8,64,65]. In addition, it has been reported that activation of cyclic electron flow (CEF) is associated with increased genotype tolerance of *Coffea* spp. [5] or tobacco [66]. However, the negative amplitude of *ΔW*_OI_ (*W*_OI_ > 1) at 55 °C indicated damages to the reduction capacity of the final acceptors. When these results were related to φ_Ro_ (Figure 8, Side A; Table S2; Figure 10, step 7), they showed similar responses at 45 °C and 50 °C, but with high average values. As the double normalization of *W*_IP_ and *ΔW*_IP_ reflects the rate of reduction of final PSI acceptors [56], negative amplitudes in *ΔW*_IP_ of *C. canephora* (Figure 7) indicated that increases in temperatures tended to decrease the rate of reduction of the final PSI acceptors (Figure 10, step 7). This was primarily due to the blockage of electron flow along the ETC, because even with increased PSI activity, the reduction rate of final acceptors decreased.

The ABS/CS_0_ parameter is related to the absorption of light energy per unit leaf area and the functional size of the antenna (Figure 10, step 1) [38,61]. Our results indicated that clone ‘A1’ had a lower capacity for absorption of light energy by the antenna complex than clone ‘3V’, especially after water deficit (Figure 8, Side B; Table S3). The φ_Po_ parameter is related to the maximum capacity of PSII to reduce Q_A_, using the absorbed energy (Figure 10, step 4) [61]. Significant reduction of φ_Po_ in *C. canephora* was observed only at 50 °C and 55 °C, where ‘A1’ showed higher φ_Po_ values compared to ‘3V’ only at 55 °C (Figure 8, Side A; Table S2). A similar trend was observed for the TR_0_/CS_0_ parameter (Figure 8, Side B; Table S2; Figure 10, step 2), which is related to the capture of excitation energy by PSII [67]. The opposite responses in φ_Do_ (Figure 8, Side A; Table S2) and DI_0_/CS_0_ (Figure 8, Side B; Table S3) were observed (Figure 10, step 1), indicating increased energy dissipation in response to increased temperature. The increase in energy dissipation here observed is possibly associated with an enhancement of CEF reported in plants subjected to heat stress [68]. The CEF enhancement induces changes in the trans-thylakoid proton gradient [69] and, consequently, promotes the activation of the xanthophyll cycle, which plays a key role in the thermal dissipation of excess energy [70]. ‘A1’-WW and ‘A1’-WS showed lower dissipation of energy compared to ‘3V’-WW and ‘3V’-WS at 55 °C. Lower energy absorption by the antenna complex followed by its lower dissipation by PSII permits increased energy utilization efficiency and leads to a reduced overload of PSII [38,61,71]. Therefore, the higher values of φ_Po_ and TR_0_/CS_0_ at 55 °C in ‘A1’, under both WW and WS water treatments, may be related to lower energy absorption in this clone, which resulted in lower dissipation of the excitation energy than in ‘3V’.

The progressive increase in supra-optimal temperatures triggers the inactivation of PSII RCs [11] and can be monitored through the RC/CS_0_ parameter (Figure 8, Side B; Table S3; Figure 10, step 2). It describes the density of the active RCs able to reduce Q_A_, so its decrease is related to greater inactivation of RCs [61,72,73]. In *C. canephora* exposed to elevated temperatures, lower inactivation of RCs was observed in ‘A1’ (especially after water deficit experience—‘A1’-WS) than in ‘3V’ (Figure 8, Side B; Table S3). This reduced inactivation of RCs is directly related to greater efficiency in energy capture and transport, and lower non-photochemical dissipation [61,73], as observed in our results (Figure 8, Side B; Table S3).

The PI_abs_ parameter is an index of PSII performance that provides information on the components of energy absorption, capture, and transport (Figure 10, steps 1 to 5) [74]. In *C. canephora,* at temperatures above 40 °C, increased reductions in PI_abs_ values were observed in all treatments. In ‘A1’-WS, these reductions were smaller than in ‘A1’-WW, while ‘3V’-WS showed greater reductions than ‘3V’-WW. The reduced values of PI_abs_ are directly related to the level of damage to the photosynthetic apparatus, involving its structure and activity [33,61,73]. The exposure of *C. canephora* plants to recurrent cycles of water deficit may result in a possible mitigation of the effects of heat stress, especially for clone ‘A1’, supported by previously observed PI_abs_ responses, which indicated drought acclimation in the second drought event [6].

**Figure 11:**
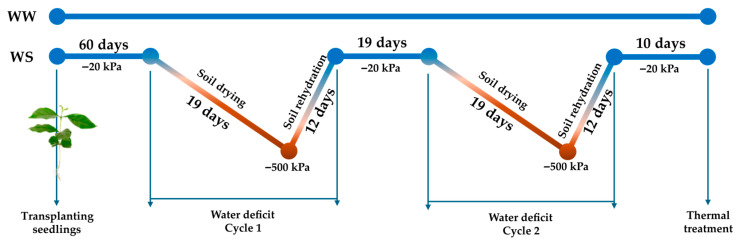
Experimental flow chart. Seedlings of *Coffea canephora* clones ‘A1’ and ‘3V’ were transplanted into 31 L pots. The plants were grown for 60 days under full irrigation (Ψ_mSoil_ ≈ −20 kPa). After this period, a group of plants was subjected to two cycles of water deficit (WS; soil drying + rehydration) until reaching Ψ_mSoil_ ≈ −500 kPa (red part of lines), and a group was kept under full irrigation (WW, blue lines). Between the two cycles of water deficit, for 19 days, the plants were kept under full irrigation (blue lines). Ten days after the end of the second cycle of water deficit, the leaves were collected and subjected to heat treatment.

Here it was demonstrated that the analysis of the OJIP kinetics, as well as the JIP_Test_, was efficient in detecting the differential response of *C. canephora* clones subjected to two recurrent water deficit cycles and, subsequently, to thermal stress. Indeed, we confirmed here our hypothesis that pre-existing water deficit would result in induction of tolerance responses of the photosynthetic apparatus to thermal stress. However, we observed that those responses were genotype dependent. Previous studies have shown that clone ‘3V’ exhibits greater stability of photochemical responses throughout two successive cycles of soil water deficit than ‘A1’, as well as a slower decline in leaf transpiration, thereby maintaining a higher leaf cooling capacity during the first stress cycle [6]. This is largely due to its greater root deepening capacity [45]. In contrast, during the first cycle of water deficit, clone ‘A1’ exhibits more severe photochemical damage, which later resembled the responses observed in ‘3V’, suggesting a possible acclimation of PSII to stress conditions [6]. Specifically, our results suggested that clone ‘A1’ seemed to have acquired tolerance through previous drought stress (priming effect), while clone ‘3V’ appears to be more sensitive to supra-optimal temperatures, which followed two water deficit cycles. We observed Ψ_mSoil_ values of ca. 15% lower in ‘3V’-WS when compared to ‘A1’-WS (Figure 11), but we suggest that this increased sensitivity in ‘3V’ was inherent to the clone and not due to the small difference in Ψ_mSoil_. Furthermore, the selection of more sensitive variables, such as *ΔW*_OJ_, *ΔW*_OI_ (*W*_OI_ < 1), *ΔW*_IP_, φ_Po_, φ_Eo_, RC/CS_0_ and PI_abs_, for the detection of the acquisition of tolerance to heat stress is recommended.

We would like to highlight that our work had more of a phenomenological than mechanistic proposition, discovering changes in ChF patterns related to “stress memory” phenomena in coffee plants. Despite this, our results indicated potential for further exploration of the mechanisms involved in acquiring tolerance in different genotypes, and for deciphering the possibilities of improving *Coffea* spp. priming techniques. Therefore, we are deducing that the acclimation of PSII in clone ‘A1’ under water deficit conditions (“stress memory”) was the main factor underlying its greater thermotolerance. The deep confirmation of “stress memory” mechanisms involves alterations in antioxidant metabolism and transcriptional memory, mainly related to the expression of genes linked to ABA synthesis, the encoding of MYB signaling proteins and miRNAs [75]. Additionally, lipid metabolism and expression of genes linked to aquaporins, chaperonins, 70 kDa heat shock proteins, and antioxidant enzymes are also related to this phenomenon [46].

## 4. Materials and Methods

### 4.1. Plant Material and Growing Conditions

Experiments were conducted in a greenhouse at the State University of Northern Rio de Janeiro, in Campos dos Goytacazes (21°44′47″ S, 41°18′24″ W, at 10 m a.s.l), RJ, Brazil. Fourteen plants of two *C. canephora* cv. Conilon clones, designated as ‘3V’ and ‘A1’, were cultivated. The ‘3V’ clone was previously characterized to have deeper root growth [45], root architecture over soil layers [76], and tolerance to water deficit, considering the dynamics of some physiological [6], anatomical, and morphological [45] parameters. The ‘A1’ clone had been characterized to have shallower root growth [76] and to be more sensitive to water deficit, considering the dynamics of some physiological [6], but more conservative in water use, considering several anatomical and morphological parameters [45].

Five-month-old vegetatively propagated seedlings grown under non-limited water conditions were transplanted into polyvinyl chloride (PVC) tubes with a capacity of approximately 31 L, filled with a substrate composed of red-yellow latosol and sand in a 4:1 ratio, which was corrected with limestone and fertilized. The plants were irrigated daily to maintain soil water retention capacity for 60 days. After this period, seven plants of each clone were kept under full irrigation (WW, i.e., well-watered), while the other seven were subjected to two successive cycles of soil drying and rehydration (WS; water deficit treatment), as in the scheme (Figure 11). The treatments were designated as ‘3V’-WW and ‘A1’-WW, and ‘3V’-WS and ‘A1’-WS.

Soil matric potential (Ψ_mSoil_) was monitored daily using Teros-21 sensors, and the data were stored in ZL6 Pro dataloggers (METER Group, Pullman WA, USA). The sensors were positioned at a soil depth of 0.1 m and 0.5 m. During the two soil drying cycles, plants were not irrigated until Ψ_mSoil_ values dropped below −500 kPa. Subsequently, the soil was re-irrigated to allow plant recovery. Each cycle of soil drying and rehydration lasted 31 days, with a 19-day recovery period between the cycles and a final recovery period of 10 days.

The plants were cultivated in a randomized block design. Throughout the experimental period, variables such as air temperature (T_air_, °C), relative humidity (RH, %), and photosynthetic photon flux density (PPFD, in µmol photons m^−2^ s^−1^) were monitored daily using a Weather Station Watchdog 2000 (Spectrum Technologies, Plainfield, IL, USA). The vapor pressure deficit of the air (VPD_air_, kPa) was calculated based on air temperature and humidity values, as proposed by [77].

During the experimental period, the PPFD reached maximum values of 1291.7 ± 32.1 µmol photons m^−2^ s^−1^ and average values of 627.6 ± 19.3 µmol photons m^−2^ s^−1^. RH varied between 41.4 ± 0.9% (minimum) and 89.9 ± 0.2% (maximum), with a mean of 71.2 ± 0.5%. T_air_ fluctuated from 22.8 ± 0.2 °C (minimum) to 41.6 ± 0.4 °C (maximum), averaging 29.7 ± 0.2 °C. The VPD_air_ spanned from 0.3 ± 0.01 kPa (minimum) to 4.9 ± 0.1 kPa (maximum), with a mean of 1.7 ± 0.1 kPa.

The minimum observed soil matric potential (Ψ_mSoil_) values for ‘3V’-WS during the first and second cycle of water deficit were −406 kPa and −446 kPa, respectively. For ‘A1’-WS, the minimum Ψ_mSoil_ values during the first and second cycles were −332 kPa and −376 kPa, respectively. In the irrigated treatments ‘3V’-WW and ‘A1’-WW, Ψ_mSoil_ was maintained at −11 kPa and −15 kPa, respectively, throughout the experimental period.

### 4.2. Thermal Treatments

For the application of thermal treatments, we followed the methodology described by [47]. Briefly, 90 days after the initiation of the first water deficit cycle, the five most vigorous plants from each of four treatments (‘3V’-WW, ‘A1’-WW, ‘3V’-WS, and ‘A1’-WS) were selected. In coffee plants, younger leaves are more sensitive to temperature increases than older ones [15]. Therefore, fully expanded leaves that had developed during the two successive cycles of soil drying and rehydration were collected at predawn. These leaves were placed in dark, moistened bags with wet paper towels and transported to the laboratory.

In the laboratory, under dark conditions, leaf discs (~2 cm^2^) were cut from the leaf blade, avoiding the central vein. The leaf discs from the five plants were mixed to create five random samples for each treatment (WW and WS) of each genotype (‘A1’ and ‘3V’). The leaf discs were exposed to five single short-term high temperatures (35 °C, 40 °C, 45 °C, 50 °C, and 55 °C). Short-stress exposure to temperatures starting from 35 °C was chosen, considering that *C. canephora* is not as robust as thought, being highly sensitive to temperature, surviving the mean maximum temperature of ~30 °C during vegetative growth and flowering, which strongly impacts final yield [78]. Furthermore, in our experiment, plants were grown under mean and maximum temperatures ranging between 30 and 40 °C, respectively [6]. Even at 50 and 55 °C, short-stress exposure of 15 min does not cause total loss of PSII functionality, considering some JIP_Test_ parameters in *Carica papaya* [61].

The leaf discs were placed in perforated plastic containers, immersed in a water bath and incubated independently for 15 min at each of the five temperatures. After incubation, they were removed from the water bath, dried with paper towels, and prepared for ChlF emission measurements.

### 4.3. Chlorophyll a Fluorescence Emission Measurement

To monitor ChlF emission in the leaf discs, we used a non-modulated fluorimeter model Pocket PEA (Hansatech Instruments Ltd., Pentney, King’s Lynn, UK) equipped with a leaf clip. Typically, leaf clips are employed to adapt the leaf to darkness, allowing for the complete oxidation of PSII RCs (“open” RCs) and preparing them to receive electrons. Since the entire procedure was conducted in the dark, the dark adaptation step was not necessary.

To induce the ChlF emission curve (the OJIP transient), a pulse of saturating red light (~627 nm) with an intensity of 3500 µmol photons m^−2^ s^−1^ was applied to the leaf discs. The emitted ChlF was detected by a PIN photodiode, amplified, and recorded at intervals of 10 µs up to 2 ms, 100 µs between 2 and 3 ms, 1 ms between 3 and 30 ms, 10 ms between 30 and 300 ms, and 100 ms between 300 and 1000 ms. Data were recorded over a period of 1 s. Additionally, the basic ChlF parameters recorded at 20 µs (*F*_0_—initial fluorescence), 150 µs (*F*_L_—L phase), 300 µs (*F*_300µs_), 2 ms (*F*_J_—J phase), 30 ms (*F*_I_—I phase), and 300 ms (*F*_P_~*F*_M_, where *F*_M_ is the maximum fluorescence after the saturating light pulse) were used to calculate the JIP_Test_ parameters [48,49,51] using the PEA Plus software 1.0 (Hansatech Instruments Ltd.). Equations and definitions of JIP_Test_ parameters obtained from chlorophyll *a* fluorescence emission (O-J-I-P) analyses are shown in Table S5.

### 4.4. Membrane Permeability

Ten different leaf discs (63.62 mm^2^) were removed along the leaf blade, avoiding the midrib. These discs were washed in distilled water and placed in test tubes with 10 mL of distilled water. The tubes were partially immersed in a water bath previously adjusted to different temperatures (35 °C, 40 °C, 45 °C, 50 °C and 55 °C) and incubated for 15 min. Two hours after removal from the water bath and stabilization, the electrical conductivity of the solution was measured with an Edge^EC^ conductivity meter (model HI2003-02, Hanna Instruments, Barueri, SP, Brazil). Subsequently, the tubes were kept for 2 h in a water bath at 90 °C, and after cooling, the total conductivity of the solution was measured again. The membrane permeability was calculated as the percentage of the total conductivity for each temperature.

### 4.5. Data Processing and Analysis

For the OJIP transient kinetics analysis, the recorded ChlF data over the induction time were plotted on a logarithmic scale. Subsequently, the data were double-normalized between the O and P steps as *W*_OP_ = (*F*_t_ − *F*_O_)/(*F*_P_ − *F*_O_) and *ΔW*_OP_ = (*W*_OP(Temperature)_ − *W*_OP(35°C)_); between the O and J steps as *W*_OJ_ = (*F*_t_ − *F*_O_)/(*F*_J_ − *F*_O_) and *ΔW*_OJ_ = (*W*_OJ(Temperature)_ − *W*_OJ(35°C)_); between the O and K steps as *W*_OK_ = (*F*_t_ − *F*_O_)/(*F*_K_ − *F*_O_) and *ΔW*_OK_ = (*W*_OK(Temperature)_ − *W*_OK(35°C)_); between the O and I steps as *W*_OI_ = (*F*_t_ − *F*_O_)/(*F*_I_ − *F*_O_) and *ΔW*_OI_ = (*W*_OI(Temperature)_ − *W*_OI(35°C)_), for 1 < *W*_OI_ > 1; and between the I and P steps as *W*_IP_ = (*F*_t_ − *F*_I_)/(*F*_P_ − *F*_I_) and *ΔW*_IP_ = (*W*_IP(Temperature)_ − *W*_IP(35°C)_) (Table S5). Each plotted curve represents an average of five repetitions, each corresponding to a leaf disc. Data normalization was performed using Excel^®^, and graphs were plotted using OriginPro, version 2016^®^ (OriginLab Corporation, Northampton, MA, USA).

For the JIP_Test_ and membrane permeability data, a two-way analysis of variance (ANOVA) with a completely randomized design (4 × 5) with five replications was performed. The sample size was considered satisfactory due to meticulous collection of leaves of the same age and position on plagiotropic axes. The ANOVA included two factors: (1) treatments of clones and water regimes (‘3V’-WW, ‘A1’-WW, ‘3V’-WS, and ‘A1’-WS) and (2) temperatures (35 °C, 40 °C, 45 °C, 50 °C, and 55 °C). Such analysis was conducted using R software, version 4.4.0 [79]. In the case of statistical significance (*p*-value < 0.05), means were compared using the Tukey test at a 5% significance level using the ‘agricolae’ package, version 1.3-7.

## 5. Conclusions

The selection of genotypes with tolerance to water and heat stress is an important tool for the more resilient cultivation of *C. canephora* to environmental stresses. We observed that the imposition of supra-optimal temperatures through heat shock in *C. canephora* clones ‘3V’ and ‘A1’ caused changes in the kinetics of the OJIP phase of a Chl *a* fluorescence transient, indicating damage to the photosynthetic apparatus, specifically in PSII. The occurrence of two recurrent cycles of water deficit before heat stress appeared to act as a priming effect in the ‘A1’ clone, making it much more tolerant to temperature increases. In contrast, the ‘3V’ clone was more sensitive to supra-optimal temperatures, both with and without previous water deficit. The results were observed through changes in the kinetics of the OJIP phase of Chl *a* fluorescence induction, as well as the quantitative analysis of the JIP_Test_. Complete loss of the typical OJIP transient shape was observed in clone ‘3V’ after short-term heat shock at the highest temperature (55 °C), whereas clone ‘A1’ maintained the characteristic curve pattern, particularly after the occurrence of water deficit stress. These responses were reflected in JIP-test parameters such as ET_0_/CS_0_, RC/CS_0_, and PI_abs_, in which clone ‘A1’, especially after two water deficit cycles, maintained significantly higher values compared to the other treatments. We concluded that the increase in heat tolerance promoted by water deficit is genotype-dependent.

## Data Availability

The raw data supporting the conclusions of this article will be made available by the authors on request.

## Supplementary Materials

The following supporting information can be downloaded at: https://www.mdpi.com/article/10.3390/plants15050740/s1, Table S1: Summary of ANOVA showing *p*-values for parameters derived from the JIP_Test_, for treatments ‘3V’-WW, ‘3V’-WS, ‘A1’-WW, and ‘A1’-WS incubated at different temperatures (35 °C, 40 °C, 45 °C, 50 °C, and 55 °C) for 15 min; Table S2: The quantum efficiency or the energy flux ratio parameters derived from the analysis of OJIP transients of Chl a fluorescence using the JIP_Test_, for ‘3V’-WW, ‘3V’-WS, ‘A1’-WW, and ‘A1’-WS incubated at different temperatures (35 °C, 40 °C, 45 °C, 50 °C, and 55 °C) for 15 min. Note: φ_Po_ represents the maximum quantum yield of primary PSII photochemistry; Ψ_Eo_, the probability with which a PSII-trapped electron is transferred from Q_A_ to Q_B_; φ_Eo_, the quantum yield of electron transport flux from Q_A_ to Q_B_; φ_Do_, the quantum yield of energy dissipation; δ_Ro_, the probability with which an electron from Q_B_ is transferred until it reaches the PSI electron acceptor(s); φ_Ro_, the quantum yield of electron transport flux until it reaches the PSI electron acceptors; Table S3: Phenomenological energy flux parameters and photosynthetic indices derived from the analysis of OJIP transients using JIP_Test_, for ‘3V’-WW, ‘3V’-WS, ‘A1’-WW, and ‘A1’-WS incubated at different temperatures (35 °C, 40 °C, 45 °C, 50 °C, and 55 °C) for 15 min. ABS/CS_0_, absorbed energy; TR_0_/CS_0_, energy flux trapped by PSII reaction centers; ET_0_/CS_0_, electron transfer through PSII; DI_0_/CS_0_, thermal dissipation of energy in PSII; RC/CS_0_, density of reaction centers capable of Q_A_ reduction, described per unit of leaf cross-section (CS). SFI_abs_, structure-function index; PI_abs_, PSII performance index normalized by absorbed energy; Table S4: Technical parameters of ChlF derived from the analysis of OJIP transients, for ‘3V’-WW, ‘3V’-WS, ‘A1’-WW, and ‘A1’-WS incubated at different temperatures (35 °C, 40 °C, 45 °C, 50 °C, and 55 °C) for 15 min. *V*_K_, relative variable fluorescence at the K-step; *V*_J_, relative variable fluorescence at the J-step; *V*_I_, relative variable fluorescence at the I-step; *W*_L_, relative variable fluorescence at the L-step to the amplitude *F*_J_ − *F*_O_; *W*_K_, relative variable fluorescence at the K-step to the amplitude *F*_J_ − *F*_O_; OEC, the fraction of oxygen-evolving complexes (OEC) centers; *F*_K_/*F*_J_, indicator of electron donation limitations on the donor side of PSII; Table S5: Equations and definitions of JIP_Test_ parameters obtained from chlorophyll *a* fluorescence emission (O-J-I-P) analyses.

## Abbreviations

The following abbreviations are used in this manuscript:

ADP	Adenosine diphosphate
ATP	Adenosine triphosphate
Chl	Chlorophyll
ChlF	Chlorophyll fluorescence
Cyt	Cytochrome
ETC	Electron-transport chain
FD	Ferredoxin
FRN	Ferredoxin-NADP^+^ reductase
H^+^	Hydrogen proton
LHCI	Light-harvesting complex of photosystem I
LHCII	Light-harvesting complex of photosystem II
NADPH	Reduced nicotinamide adenine dinucleotide phosphate
NADP^+^	Oxidized nicotinamide adenine dinucleotide phosphate
OEC	Oxygen-evolving complex
PC	Plastocyanin
Pheo	Pheophytin
Pi	Inorganic phosphate
PPFD	Photosynthetic photon flux density
PQ	Plastoquinone
PQH_2_	Plastohydrolquinol
PSI	Photosystem I (P700)
PSII	Photosystem II (P680)
PVC	Polyvinyl chloride
Q_A_	Quinone A
Q_B_	Quinone B
RC	Reaction center
RH	Relative humidity
T_air_	Air temperature
VPD_air_	Vapor pressure deficit of the air
WS	Water stress
WW	Well-watered
Ψ_mSoil_	Soil matric potential
