# Supplementary material for: The OJIP Kinetics Analysis Reveals Differential Thermal Tolerance Responses in Photosystem II of Coffea canephora Clones After Two Recurrent Cycles of Water Deficit
Source: Plants (Basel). 2026 Feb 28;15(5):740. doi: 10.3390/plants15050740 (PMC12986973; doi:10.3390/plants15050740)
Supplement: Supplementary file 1 [file plants-15-00740-s001.zip › plants-4129813-supplementary.pdf]

plants

MDPI

Supplementary materials for:

Article

The OJIP Kinetics Analysis Reveals Differential Thermal Tolerance Responses in Photosystem II of *Coffea canephora* Clones After Two Recurrent Cycles of Water Deficit

Guilherme Augusto Rodrigues de Souza [1,2,*], Danilo Força Baroni [1], Diesily Andrade Neves [1], Anne Reis Santos [1], Laísa Zanelato Correia [1], Larissa Crisostomo de Souza Barcellos [1], Ellen Moura Vale [1], Wallace de Paula Bernado [3], Weverton Pereira Rodrigues [4], Antelmo Ralph Falqueto [5], Miroslava Rakocevic [1,6,*], Eliemar Campostrini [1]

[1] Plant Physiology Laboratory (LMGV), State University of North Fluminense Darcy Ribeiro (UENF), 2000 Alberto Lamego Ave., Parque Califórnia, Campos dos Goytacazes 28013-602, RJ, Brazil; baronidf@gmail.com (D.F.B.); diesilyandrade@gmail.com (D.A.N.); annersantos@outlook.com (A.R.S.); laisazanelatocorreia@gmail.com (L.Z.C.); lbarcellos.uenf@gmail.com (L.C.d.S.B.); ellenmoura27@gmail.com (E.M.V.); campostenator@gmail.com (E.C.)

[2] Instituto de Biodiversidade e Sustentabilidade, Universidade Federal do Rio de Janeiro (UFRJ), 764, Amaro Reinaldo dos Santos Silva Ave., São José Barreto, Macaé 27965-045, RJ, Brazil

[3] Sylvio Moreira Citrus Research Center-Agronomic Institute (IAC), Rod. Anhangüera, km 158, Cascalho, Cordeirópolis 13490-000,, SP, Brazil; wallace-bernardo@hotmail.com

[4] Centro de Ciências Agrárias, Universidade Estadual da Região Tocantina do Maranhão (UEMASUL), 100 Agrária Ave., Res. Colina Park, Imperatriz 65900-001, MA, Brazil; wevertonuenf@hotmail.com

[5] Departamento de Ciências Agrárias e Biológicas, Universidade Federal do Espírito Santo (UFES), BR 101 Norte, km 60, Bairro Litorâneo, CEP, São Mateus 29932-540, ES, Brazil; antelmofalqueto@gmail.com

[6] Laboratory of Crop Physiology, Department of Plant Biology, Institute of Biology, State University of Campinas (UNICAMP), Campinas 13083-862, SP, Brazil

* Correspondence: guilherme.rodrigues@edu.uniube.br (G.A.R.d.S.); mima.rakocevic61@gmail.com (M.R.).

Academic Editor: Firstname Lastname

Received: date
Revised: date
Accepted: date
Published: date

Citation: To be added by editorial staff during production.

Copyright: © 2025 by the authors. Submitted for possible open access publication under the terms and conditions of the Creative Commons Attribution (CC BY) license (https://creativecommons.org/licenses/by/4.0/).

plants

MDPI

Table S1 Summary of ANOVA showing *p*-values for parameters derived from the JIP$_{Test}$, for treatments '3V'-WW, '3V'-WS, 'A1'-WW, and 'A1'-WS incubated at different temperatures (35 °C, 40 °C, 45 °C, 50 °C, and 55 °C) for 15 min.

Sources of variation	Parameters (*P* - value)						
		F_0	F_L	F_K	F_J	F_I	F_M
**Treatment x Temperature**		<0.0001	<0.0001	<0.0001	<0.0001	<0.0001	0.0001
**Treatment**		0.0025	0.0001	<0.0001	<0.0001	0.0013	0.9993
**Temperature**		<0.0001	<0.0001	<0.0001	0.0345	<0.0001	<0.0001
		φ_{Po}	Ψ_{Eo}	φ_{Eo}	φ_{Do}	δ_{Ro}	φ_{Ro}
**Treatment x Temperature**		<0.0001	0.0829	<0.0001	<0.0001	0.6096	<0.0001
**Treatment**		0.0066	<0.0001	<0.0001	0.0066	0.3252	<0.0001
**Temperature**		<0.0001	0.0048	<0.0001	<0.0001	0.0069	<0.0001
	ABS/CS_0	TR_0/CS_0	ET_0/CS_0	DI_0/CS_0	RC/CS_0	SFI_{abs}	PI_{abs}
**Treatment x Temperature**	<0.0001	<0.0001	<0.0001	<0.0001	<0.0001	<0.0001	<0.0001
**Treatment**	0.0007	<0.0001	<0.0001	0.0007	<0.0001	<0.0001	<0.0001
**Temperature**	<0.0001	<0.0001	<0.0001	<0.0001	<0.0001	<0.0001	<0.0001
	V_K	V_J	V_I	W_L	W_K	OEC	F_K/F_J
**Treatment x Temperature**	0.0037	0.0005	<0.0001	<0.0001	<0.0001	<0.0001	<0.0001
**Treatment**	0.0222	0.0061	0.0005	0.0092	0.0153	0.0979	0.0084
**Temperature**	0.776	0.9435	0.5639	<0.0001	<0.0001	<0.0001	<0.0001

Red values indicate significant statistical difference for the source of variation to each parameter (*P* - value < 0.05).

Table S2 The quantum efficiency or the energy flux ratio parameters derived from the analysis of OJIP transients of Chl a fluorescence using the JIP_{Test}, for '3V'-WW, '3V'-WS, 'A1'-WW, and 'A1'-WS incubated at different temperatures (35 °C, 40 °C, 45 °C, 50 °C, and 55 °C) for 15 min. Note: φ_{Po} represents the maximum quantum yield of primary PSII photochemistry; Ψ_{Eo}, the probability with which a PSII-trapped electron is transferred from Q_A to Q_B; φ_{Eo}, the quantum yield of electron transport flux from Q_A to Q_B; φ_{Do}, the quantum yield of energy dissipation; δ_{Ro}, the probability with which an electron from Q_B is transferred until it reaches the PSI electron acceptor(s); φ_{Ro}, the quantum yield of electron transport flux until it reaches the PSI electron acceptors.

Parameter	Treatment	Temperature					
		35 °C	40 °C	45 °C	50 °C	55 °C	Means
φ_{Po}	'3V'-WW	0.75 aA	0.71 aA	0.70 aA	0.51 aB	0.05 bC	0.55 b
	'3V'-WS	0.75 aA	0.76 aA	0.70 aAB	0.58 aB	0.01 bC	0.56 b
	'A1'-WW	0.71 aA	0.70 aA	0.68 aA	0.48 aB	0.35 aB	0.59 ab
	'A1'-WS	0.76 aA	0.76 aA	0.73 aA	0.49 aB	0.37 aB	0.62 a
	Means	0.74 A	0.74 A	0.70 A	0.51 B	0.20 C	
Ψ_{Eo}	'3V'-WW	0.53 aA	0.46 aA	0.56 aA	0.43 aA	0.32 aA	0.46 b
	'3V'-WS	0.58 aA	0.61 aA	0.64 aA	0.57 aA	0.57 aA	0.59 a
	'A1'-WW	0.49 aA	0.49 aA	0.49 aA	0.51 aA	0.36 aA	0.47 b
	'A1'-WS	0.61 aA	0.62 aA	0.65 aA	0.57 aA	0.66 aA	0.62 a
	Means	0.56 AB	0.55 AB	0.59 A	0.52 AB	0.48 B	
φ_{Eo}	'3V'-WW	0.40 abA	0.34 bA	0.39 abA	0.22 bB	0.02 bC	0.27 c
	'3V'-WS	0.44 abAB	0.46 aA	0.45 aA	0.33 aB	0.003 bC	0.34 b
	'A1'-WW	0.35 bA	0.34 bA	0.34 bA	0.25 abA	0.14 aB	0.29 c
	'A1'-WS	0.47 aA	0.48 aA	0.47 aA	0.28 abB	0.24 aB	0.39 a
	Means	0.41 A	0.40 A	0.41 A	0.27 B	0.10 C	
φ_{Do}	'3V'-WW	0.25 aC	0.29 aC	0.30 aC	0.49 aB	0.95 aA	0.46 a
	'3V'-WS	0.25 aC	0.24 aC	0.30 aBC	0.42 aB	0.99 aA	0.44 a
	'A1'-WW	0.29 aB	0.30 aB	0.32 aB	0.52 aA	0.65 bA	0.42 ab
	'A1'-WS	0.24 aB	0.24 aB	0.28 aB	0.51 aA	0.63 bA	0.38 b
	Means	0.26 C	0.27 C	0.30 C	0.49 B	0.80 A	
δ_{Ro}	'3V'-WW	0.28 aA	0.31 aA	0.75 aA	0.85 aA	1.15 aA	0.67 a
	'3V'-WS	0.37aA	0.34 aA	0.73 aA	1.05 aA	1.99 aA	0.89 a
	'A1'-WW	0.32 aA	0.35 aA	0.45 aA	0.68 aA	0.22 aA	0.41 a
	'A1'-WS	0.35 aA	0.39 aA	0.58 aA	0.59 aA	2.34 aA	0.85 a
	Means	0.33 B	0.35 B	0.63 AB	0.79 AB	1.43 A	
φ_{Ro}	'3V'-WW	0.11 aB	0.11 aB	0.29 aA	0.19 bB	0.01 bC	0.14 b
	'3V'-WS	0.16 aB	0.16 aB	0.32 aA	0.35 aA	0.01 bC	0.19 a
	'A1'-WW	0.11 aAB	0.12 aAB	0.15 bA	0.18 bA	0.05 abB	0.12 b
	'A1'-WS	0.16 aB	0.19 aAB	0.27 aA	0.18 bAB	0.13 aB	0.19 a
	Means	0.14 B	0.14 B	0.26 A	0.23 A	0.05 C	

The estimated means for each treatment are shown (n = 5). P-value < 0.05 was considered significant. Lowercase letters indicate differences among treatments for each temperature, while the uppercase letters indicate differences among temperatures for each treatment, according to the Tukey test at 95% of confidence.

Table S3 Phenomenological energy flux parameters and photosynthetic indices derived from the analysis of OJIP transients using JIP_{Test}, for '3V'-WW, '3V'-WS, 'A1'-WW, and 'A1'-WS incubated at different temperatures (35 °C, 40 °C, 45 °C, 50 °C, and 55 °C) for 15 min. ABS/CS_0, absorbed energy; TR_0/CS_0, energy flux trapped by PSII reaction centers; ET_0/CS_0, electron transfer through PSII; DI_0/CS_0, thermal dissipation of energy in PSII; RC/CS_0, density of reaction centers capable of Q_A reduction, described per unit of leaf cross-section (CS). SFI_{abs}, structure-function index; PI_{abs}, PSII performance index normalized by absorbed energy.

**Parameter**	**Treatment**	**Temperature**					
		**35 °C**	**40 °C**	**45 °C**	**50 °C**	**55 °C**	**Means**
**ABS/CS_0**	**'3V'-WW**	8392.60 aC	8738.20 aC	9016.00 aC	12662.20 aB	19739.60 bA	11709.72 a
	**'3V'-WS**	7639.60 aB	7600.80 aB	9356.80 aB	10266.20 aB	23038.00 aA	11580.28 a
	**'A1'-WW**	8624.20 aC	8943.80 aC	9855.60 aBC	12781.00 aB	16631.80 cA	11367.28 a
	**'A1'-WS**	7735.40 aC	7889.00 aBC	8651.60 aBC	13103.00 aA	11141.60 dAB	9704.12 b
	**Means**	8097.95 C	8292.95 C	9220.00 C	12203.10 B	17637.75 A	
**TR_0/CS_0**	**'3V'-WW**	6301.85 aA	6214.69 aA	6337.67 aA	6381.49 aA	964.96 bB	5240.13 bc
	**'3V'-WS**	5743.22 aA	5790.85 aA	6472.87 aA	5961.78 aA	150.79 bB	4823.91 c
	**'A1'-WW**	6116.47 aAB	6285.96 aAB	6729.75 aA	6038.36 aAB	4882.09 aB	6010.53 a
	**'A1'-WS**	5880.77 aA	6012.08 aA	6266.27 aA	6267.92 aA	3721.96 aB	5629.80 ab
	**Means**	6010.58 A	6075.90 A	6451.64 A	6162.39 A	2429.95 B	
**ET_0/CS_0**	**'3V'-WW**	3313.21 aA	2917.74 aA	3512.52 aA	2745.16 aA	380.24 bB	2573.77 b
	**'3V'-WS**	3347.42 aA	3489.57 aA	4146.22 aA	3395.96 aA	79.45 bB	2891.72 b
	**'A1'-WW**	3034.40 aA	3077.89 aA	3348.56 aA	3114.16 aA	1855.98 aB	2886.20 b
	**'A1'-WS**	3602.86 aA	3735.38 aA	4039.67 aA	3601.48 aA	2367.08 aB	3469.30 a
	**Means**	3324.47 AB	3305.15 AB	3761.74 A	3214.19 B	1170.69 C	
**DI_0/CS_0**	**'3V'-WW**	2090.76 aB	2523.50 aB	2678.33 aB	6280.71 aB	18774.64 bA	6469.59 a
	**'3V'-WS**	1896.38 aB	1809.95 aB	2883.93 aB	4304.42 aB	22887.21 aA	6756.38 a
	**'A1'-WW**	2507.73 aB	2657.85 aB	3125.85 aB	6742.64 aB	11749.70 cA	5356.75 ab
	**'A1'-WS**	1854.63 aB	1876.92 aB	2385.33 aB	6835.08 aA	7419.64 dA	4074.32 b
	**Means**	2087.37 C	2217.05 C	2768.36 C	6040.71 B	15207.80 A	
**RC/CS_0**	**'3V'-WW**	7476.30 aA	5865.72 bcB	4720.02 aB	2517.65 abC	709.82 bD	4257.90 bc
	**'3V'-WS**	7308.72 aA	7760.59 aA	4890.05 aB	3120.03 abC	43.65 bD	4624.61 b
	**'A1'-WW**	5342.02 bA	5136.70 cA	4399.97 aA	2117.97 bB	2263.18 aB	3851.97 c
	**'A1'-WS**	7873.84 aA	7079.50 abA	5385.53 aB	3527.75 aC	2816.76 aC	5336.68 a
	**Means**	7000.22 A	6460.63 A	4848.89 B	2820.85 C	1458.35 D	
**SFI_{abs}**	**'3V'-WW**	3.55 bA	2.28 bB	2.08 bcB	0.45 aC	0.02 bC	1.67 b
	**'3V'-WS**	4.19 abA	4.71 aA	2.45 abB	1.03 aC	0.0001 bD	2.48 a
	**'A1'-WW**	2.18 cA	2.01 bA	1.56 cA	0.44 aB	0.27 abB	1.29 c
	**'A1'-WS**	4.79 aA	4.28 aA	2.93 aB	0.75 aC	0.90 aC	2.73 a
	**Means**	3.68 A	3.32 A	2.26 B	0.67 C	0.29 C	
**PI_{abs}**	**'3V'-WW**	31.47 bA	15.47 bB	16.50 bB	1.82 aBC	0.04 aC	13.06 b
	**'3V'-WS**	41.82 abA	51.30 aA	24.04 abB	6.01 aC	0.0002 aC	24.63 a
	**'A1'-WW**	15.02 cA	13.77 bA	10.33 bA	2.10 aA	0.95 aA	8.43 b
	**'A1'-WS**	54.55 aA	49.04 aA	30.84 aB	3.91 aC	6.48 aC	28.97 a
	**Means**	35.71 A	32.40 A	20.43 B	3.46 C	1.87 C	

The estimated means for each treatment are shown (n = 5). P-value < 0.05 was considered significant. Lowercase letters indicate differences among treatments for each temperature, while the uppercase letters indicate differences among temperatures for each treatment, according to the Tukey test at 95% of confidence.

Table S4 Technical parameters of ChlF derived from the analysis of OJIP transients, for ‘3V’-WW, ‘3V’-WS, ‘A1’-WW, and ‘A1’-WS incubated at different temperatures (35 °C, 40 °C, 45 °C, 50 °C, and 55 °C) for 15 min. V_K, relative variable fluorescence at the K-step; V_J, relative variable fluorescence at the J-step; V_I, relative variable fluorescence at the I-step; W_L, relative variable fluorescence at the L-step to the amplitude $F_J - F_O$; W_K, relative variable fluorescence at the K-step to the amplitude $F_J - F_O$; OEC, the fraction of oxygen-evolving complexes (OEC) centers; F_K/F_J, indicator of electron donation limitations on the donor side of PSII.

Parameter	Treatment	Temperature					
		35 °C	40 °C	45 °C	50 °C	55 °C	Means
V_K	'3V'-WW	0.11 aA	0.16 aA	0.17 aA	0.41 aA	1.50 aA	0.47 a
	'3V'-WS	0.09 aA	0.08 aA	0.15 aA	0.24 aA	-2.29 bB	-0.35 b
	'A1'-WW	0.16 aA	0.18 aA	0.22 aA	0.39 aA	0.41 aA	0.27 ab
	'A1'-WS	0.08 aA	0.09 aA	0.12 aA	0.23 aA	0.06 aA	0.12 ab
	Means	0.11A	0.13A	0.16A	0.32A	-0.08A	
V_J	'3V'-WW	0.48 aB	0.54 aB	0.46 aB	0.61 aAB	2.04 aA	0.83 a
	'3V'-WS	0.43 aA	0.40 aA	0.38 aA	0.47 aA	-1.83 cB	-0.03 b
	'A1'-WW	0.52 aA	0.52 aA	0.52 aA	0.52 aA	0.71 abA	0.56 ab
	'A1'-WS	0.39 aA	0.38 aA	0.37 aA	0.47 aA	0.26 bA	0.38 ab
	Means	0.46A	0.46A	0.43A	0.52A	0.29A	
V_I	'3V'-WW	0.86 aB	0.86 aB	0.59 aB	0.66 aB	1.98 aA	0.99 a
	'3V'-WS	0.80 aA	0.80 aA	0.56 aA	0.44 aA	-0.63 cB	0.39 b
	'A1'-WW	0.85 aA	0.83 aA	0.78 aA	0.67 aA	0.96 bA	0.82 a
	'A1'-WS	0.79 aA	0.76 aA	0.64 aA	0.69 aA	0.60 bA	0.69 ab
	Means	0.83A	0.81A	0.64A	0.62A	0.73A	
W_L	'3V'-WW	0.10 aC	0.13 aBC	0.17 aBC	0.35 aAB	0.51 bA	0.26 ab
	'3V'-WS	0.10 aB	0.09 aB	0.18 aB	0.25 aB	1.12 aA	0.35 a
	'A1'-WW	0.14 aB	0.16 aB	0.20 aAB	0.43 aA	0.35 bAB	0.26 ab
	'A1'-WS	0.09 aB	0.11 aB	0.15 aB	0.26 aB	0.51 bA	0.22 b
	Means	0.11 C	0.12 C	0.18 C	0.32 B	0.62 A	
W_K	'3V'-WW	0.23 aB	0.29 aB	0.37 aB	0.68 abA	0.61 bA	0.44 ab
	'3V'-WS	0.22 aC	0.21 aC	0.37 aBC	0.51 bB	1.09 aA	0.48 a
	'A1'-WW	0.31 aC	0.34 aC	0.42 aBC	0.77 aA	0.60 Bab	0.49 a
	'A1'-WS	0.21 aC	0.23 aC	0.32 aBC	0.49 bAB	0.63 bA	0.38 b
	Means	0.24 D	0.27 CD	0.37 C	0.61 B	0.73 A	
OEC	'3V'-WW	1.00 aA	0.92 aA	0.83 aA	0.42 abB	0.50 aB	0.74
	'3V'-WS	1.00 aA	1.02 aA	0.80 aAB	0.62 aB	-0.11 bC	0.67
	'A1'-WW	1.00 aA	0.97 aA	0.85 aAB	0.34 bC	0.58 aBC	0.75
	'A1'-WS	1.00 aA	0.97 aA	0.86 aAB	0.64 aBC	0.47 aC	0.79
	Means	1.00 A	0.97 AB	0.83 B	0.51 C	0.36 C	
F_K/F_J	'3V'-WW	0.54 aD	0.58 abCD	0.66 aC	0.87abB	0.98 aA	0.73 ab
	'3V'-WS	0.55 aD	0.55 bD	0.70 aC	0.81 bB	1.00 aA	0.72 ab
	'A1'-WW	0.61 aB	0.62 aB	0.68 aB	0.92 aA	0.89 bA	0.74 a
	'A1'-WS	0.55 aC	0.57 abC	0.66 aB	0.85 abA	0.86 bA	0.69 b
	Means	0.56 D	0.58 D	0.68 C	0.86 B	0.93 A	

The estimated means for each treatment are shown (n = 5). P-value < 0.05 was considered significant. Lowercase letters indicate differences among treatments for each temperature, while the uppercase letters indicate differences among temperatures for each treatment, according to the Tukey test at 95% of confidence.

Table S5 Equations and definitions of JIP_{Test} parameters obtained from chlorophyll *a* fluorescence emission (O-J-I-P) analyses [1–3].

**Parameter**	**Description**
Fluorescence technical parameter	Measured fluorescence and derived from measured data
$F_O = F_{20\,\mu s}$	minimum fluorescence emitted when all PSII reaction centers are open and recorded at t = 20 μs
$F_L = F_{150\mu s}$	fluorescence intensity at the L-step recorded at t = 150 μs
$F_K = F_{300\mu s}$	fluorescence intensity at the K-step recorded at t = 300 μs
$F_J = F_{2ms}$	fluorescence intensity at the J-step recorded at t = 2 ms
$F_I = F_{30ms}$	fluorescence intensity at the I-step recorded at t = 30 ms
$F_M = F_{300ms}$	maximum fluorescence emitted when all PSII reaction centers are closed and recorded at t = 300 ms
$V_K = (F_K - F_O)/(F_M - F_O)$	relative variable fluorescence at the K-step
$V_J = (F_J - F_O)/(F_M - F_O)$	relative variable fluorescence at the J-step
$V_I = (F_I - F_O)/(F_M - F_O)$	relative variable fluorescence at the I-step
$W_L = W_{150\mu s} = (F_{150\mu s} - F_O)/(F_J - F_O)$	relative variable fluorescence at the L-step to the amplitude $F_J - F_O$
$W_K = W_{300\mu s} = (F_{300\mu s} - F_O)/(F_J - F_O)$	relative variable fluorescence at the K-step to the amplitude $F_J - F_O$
$OEC = [1 - (V_K/V_J)]_{treatment}/[1 - (V_K/V_J)]_{control}$	the fraction of Oxygen Evolving Complexes (OEC) centers
F_K/F_J	indicator of electron donation limitations on the donor side of PSII.
Quantum yields or flux ratios	Derived from measured data
$\varphi_{Po} = TR_0/ABS = [1 - (F_O/F_M)] = F_V/F_M$	maximum quantum yield of primary photochemical reactions (at t = 0)
$\Psi_{Eo} = ET_0/TR_0 = (1 - V_J)$	probability of electron transport beyond Q_A^- to electron transport chain beyond Q_A^- (at t = 0)
$\varphi_{Eo} = ET_0/ABS = [1 - (F_O/F_M)] * \Psi_{Eo} = \varphi_{Po} * \Psi_{Eo}$	quantum efficiency of electron transfer from Q_A^- (at t = 0)
$\varphi_{Do} = 1 - \varphi_{Po} = (F_O/F_M)$	quantum efficiency of energy dissipation (at t = 0)
$\delta_{Ro} = RE_0/ET_0 = (1 - V_I)/(1 - V_J)$	probability with which the electron on intersystem carriers can reduce the terminal electron acceptors on the acceptor side of PSI
$\varphi_{Ro} = \varphi_{Po} * \Psi_{Eo} * \delta_{Ro} = RE_0/ABS = \varphi_{Po} * (1 - V_I)$	quantum yield for the reduction of terminal electron acceptors on the acceptor side of PSI
Phenomenological energy fluxes	by excited cross section (CS)
RC/CS_0	density of reaction centers capable of Q_A reduction
ABS/CS_0	energy absorbed per unit cross section at the onset of measurement (at t = 0)
TR_0/CS_0	energy flux trapped by PSII reaction centers per unit cross section (at t = 0)
ET_0/CS_0	electron flux through PSII per unit cross section (at t = 0)
DI_0/CS_0	thermal dissipation of energy in PSII per unit cross section (at t = 0)
Performance index	Parameter combination
$PI_{abs} = (RC/ABS) * [\varphi_{Po}/(1-\varphi_{Po})] * [\Psi_{Eo}/(1-\Psi_{Eo})]$	performance index. An indicator of PSII functional activity normalized to absorbed energy
Structure-function index	
$SFI_{abs} = (Chl_{RC}/Chl_{tot}) * \varphi_{Po} * \Psi_{Eo}$	provides structural and functional information about the strength of the influence of internal factors promoting reactions in PSII

References

1. 1. Strasser, R.J.; Tsimilli-Michael, M.; Srivastava, A. Analysis of the Chlorophyll a Fluorescence Transient. In *Chlorophyll a Fluorescence*; Papageorgiou, G.C., Govindjee, Eds.; Advances in Photosynthesis and Respiration; Springer Netherlands: Dordrecht, The Netherlands, 2004; pp. 321–362.
2. Stirbet, A.; Govindjee. On the Relation between the Kautsky Effect (Chlorophyll a Fluorescence Induction) and Photosystem II: Basics and Applications of the OJIP Fluorescence Transient. *J. Photochem. Photobiol. B: Biol.* **2011**, *104*, 236–257, https://doi.org/10.1016/j.jphotobiol.2010.12.010
3. Goltsev, V.N.; Kalaji, H.M.; Paunov, M.; Bąba, W.; Horaczek, T.; Mojski, J.; Kociel, H.; Allakhverdiev, S.I. Variable Chlorophyll Fluorescence and Its Use for Assessing Physiological Condition of Plant Photosynthetic Apparatus. *Russ. J. Plant Physiol.* **2016**, *63*, 869–893. https://doi.org/10.1134/S1021443716050058
